# Cytoplasmic redox imbalance in the thioredoxin system activates Hsf1 and results in hyperaccumulation of the sequestrase Hsp42 with misfolded proteins

**DOI:** 10.1091/mbc.E23-07-0296

**Published:** 2024-03-05

**Authors:** Davi Goncalves, Duong Long Duy, Sara Peffer, Kevin A. Morano

**Affiliations:** a Department of Microbiology and Molecular Genetics, McGovern Medical School at UTHealth Houston, Houston, TX 77030; b Microbiology and Infectious Disease Program, MD Anderson UTHealth Graduate School at UTHealth Houston, Houston, TX 77030; Nanyang Technological University

## Abstract

Cells employ multiple systems to maintain homeostasis when experiencing environmental stress. For example, the folding of nascent polypeptides is exquisitely sensitive to proteotoxic stressors including heat, pH, and oxidative stress, and is safeguarded by a network of protein chaperones that concentrate potentially toxic misfolded proteins into transient assemblies to promote folding or degradation. The redox environment itself is buffered by both cytosolic and organellar thioredoxin and glutathione pathways. How these systems are linked is poorly understood. Here, we determine that specific disruption of the cytosolic thioredoxin system resulted in constitutive activation of the heat shock response in *Saccharomyces cerevisiae* and accumulation of the sequestrase Hsp42 into an exaggerated and persistent juxtanuclear quality control (JUNQ) compartment. Terminally misfolded proteins also accumulated in this compartment in thioredoxin reductase (*TRR1*)-deficient cells, despite apparently normal formation and dissolution of transient cytoplasmic quality control (CytoQ) bodies during heat shock. Notably, cells lacking *TRR1* and *HSP42* exhibited severe synthetic slow growth exacerbated by oxidative stress, signifying a critical role for Hsp42 under redox-challenged conditions. Finally, we demonstrated that Hsp42 localization patterns in *trr1∆* cells mimic those observed in chronically aging and glucose-starved cells, linking nutrient depletion and redox imbalance with management of misfolded proteins via a process of long-term sequestration.

## Abstract (executive summary)

Cells employ multiple systems to ensure homeostasis during environmental stress, but how the redox environment is coordinated with protein quality control is poorly understood.

Disruption of the cytosolic thioredoxin pathway in *Saccharomyces cerevisiae* results in chronic activation of the heat shock response and hyperaccumulation of the protein sequestrase Hsp42 with misfolded proteins, similar to that observed in glucose-starved and quiescent cells.

Hsp42 is shown to be required for optimal growth during redox stress, underscoring a critical role for spatial quality control during environmental and nutritional challenge.

## INTRODUCTION

Cells respond to fluctuating external and internal environmental changes through physiological adaptation driven in part by transcriptional changes in gene expression. When the stressors are proteotoxic and impact the folding, maturation, or function of the proteome, an array of cytoprotective genes that includes protein molecular chaperones, components of the ubiquitin-proteasome system (UPS) and detoxification enzymes are produced in part through the action of the heat shock transcription factor, Hsf1 (Åkerfelt *et al.*, 2010). In the yeast *Saccharomyces cerevisiae*, such stressors include heat shock, amino acid analogues, glucose starvation, and oxidative stress (Trotter *et al.*, 2002; Verghese *et al.*, 2012; Tye and Churchman, 2021). In addition to oxidizing agents like hydrogen peroxide and superoxide anions that damage proteins, a range of thiol-reactive compounds are potent inducers of Hsf1 and the resultant heat shock response (HSR; West *et al.*, 2012). Multiple chemical mechanisms of thiol-reactive proteotoxic stress have been documented, including inappropriate disulfide bond formation, irreversible oxidation, and adduct formation on sulfur atoms within the amino acids methionine and cysteine (Dahl *et al.*, 2015; Fra *et al.*, 2017; Lévy *et al.*, 2019). The relative pKa of the cysteine thiolate anion is determined by the local microenvironment and defines the reactivity of the side chain to thiol-reactive stressors; most cysteines are nonreactive while some protein cysteines are hypersensitive to insult (Winterbourn and Hampton, 2008). For example, the heat shock protein (Hsp) 70 family of chaperones possesses multiple highly reactive cysteines whose modification inhibits chaperone function (Miyata *et al.*, 2012; Wang *et al.*, 2012; Wang and Sevier, 2016). Moreover, in yeast, the Hsp70 Ssa1 governs the response of Hsf1 to thiol-reactive stress via two cysteine triggers (C264 and C303) in the ATPase domain that result in release of the chaperone from Hsf1 intrinsically disordered domains required for three-dimensional chromatin organization and transcriptional activation (Wang *et al.*, 2012; Santiago and Morano, 2022).

The linked but independent thioredoxin and glutathione systems control the redox state of the yeast cytoplasm. Electrons produced from catabolic oxidation (i.e., glycolysis, the tricarboxylic acid cycle and the pentose phosphate pathway) are shuttled through thioredoxin (Trr1) or glutathione (Glr1) reductases to thioredoxin (Trx1/2) or glutathione (GSH), respectively, that partner with peroxiredoxins to collaboratively maintain the cytoplasm in a reduced state distinct from the oxidizing environment of the endomembrane system (endoplasmic reticulum, Golgi, and lysosome/vacuole) and the extracellular space (Le Moan *et al.*, 2006; López-Mirabal and Winther, 2008; Ayer *et al.*, 2014). The glutathione system has been defined as the first line of antioxidant defense in budding yeast, which maintain high concentrations of this small molecule to detoxify thiol-reactive agents (Spector *et al.*, 2001; Le Moan *et al.*, 2006). In contrast, the thioredoxin system plays the dominant role in maintaining cellular redox homeostasis (Trotter and Grant, 2003).

The proteome is surveilled in real time via the action of the protein quality control network, comprised primarily by chaperones that recognize inappropriately exposed hydrophobic surfaces of unassembled complex subunits or regions of misfolded proteins. Once identified, these substrates are collected into transient assemblies known as Q-, or cytoplasmic quality control (CytoQ)-bodies, which include Hsp70 chaperones (Ssa1/2/3/4) as well as Hsp40 (Sis1, Ydj1) and Hsp110 (Sse1/2) cochaperones, the disaggregase Hsp104, and other components including small heat shock proteins (sHSP; Kaganovich *et al.*, 2008; Malinovska *et al.*, 2012; Miller *et al.*, 2015b; Sathyanarayanan *et al.*, 2020). It has been recently established that at least two chaperones, Btn2 and Hsp42, function as “sequestrases” that intercalate between misfolded chaperone cargo to generate the CytoQ assemblies by virtue of intrinsically disordered/prion-like domains (Malinovska *et al.*, 2012; Miller *et al.*, 2015a; Grousl *et al.*, 2018; Shrivastava *et al.*, 2022). CytoQ assemblies fail to form in cells lacking Hsp42, underscoring the important role the sequestrase plays in shepherding misfolded proteins (Specht *et al.*, 2011; Saarikangas and Barral, 2015). Multiple CytoQ bodies typically form upon proteotoxic stress, which then resolve into one or more possible depots: the internuclear quality control compartment (INQ), the cytoplasmic, juxtanuclear quality control compartment (JUNQ), or in the case of amyloid deposits and prion-like proteins, the insoluble protein deposit (IPOD; Kaganovich *et al.*, 2008; Miller *et al.*, 2015b; Hill *et al.*, 2017; Rothe *et al.*, 2018). INQ/JUNQ are marked by substrates that are subsequently extracted by Hsp104 and either refolded or ubiquitinated as a prelude to degradation (Specht *et al.*, 2011; Mathew *et al.*, 2017). The Btn2 sequestrase contains an alpha-crystallin-like domain, localizes to the nucleoplasm and is required for INQ formation, while the classic small HSP Hsp42 appears to be restricted to the cytoplasm and promotes formation of JUNQ and IPOD (Malinovska *et al.*, 2012; Miller *et al.*, 2015, a and b). INQ and JUNQ only accumulate when protein degradation is blocked and do so as one or two large static foci in cells where model misfolded proteins fused to GFP are tracked via fluorescence microscopy (Kaganovich *et al.*, 2008; Kumar *et al.*, 2022).

In this report, we use a genetic approach to generate redox stress in yeast cells via targeted deletion of major gene products comprising the thioredoxin and glutathione pathways. Strikingly, we discovered that proteotoxic stress as reported by Hsf1 activation is only experienced by cells with a disrupted cytosolic, but not mitochondrial, thioredoxin system, with no apparent role for the glutathione pathway. We found that the sequestrase Hsp42, but not Btn2 or the other yeast sHSP Hsp26, hyperaccumulated in one to two large foci in *trr1∆* cells in a structure colocalizing with the nuclear membrane, consistent with JUNQ compartments. The foci formed in *trr1∆* cells were independent of heat shock-induced transient CytoQ bodies and did not include the Hsp104 or Ssa/Ssb Hsp70 chaperones. While protein folding and degradation were not impaired in *trr1∆* cells as reported using model substrates, permanently misfolded proteins colocalized with Hsp42, further evidence of aberrant quality control in cells lacking thioredoxin reductase function. Importantly, Hsp42 sequestration was shown to be important for optimal growth and survival in unstressed or oxidant-challenged *trr1∆* cells. Finally, Hsp42 dynamics in *trr1∆* cells were found to closely mirror localization patterns in glucose-starved and aging cells, suggesting a common underlying mechanism that connects redox balance, abundance of reducing equivalents, and spatial quality control.

## RESULTS

### Cells deficient in the thioredoxin system exhibit constitutive activation of the HSR

**FIGURE 1:**
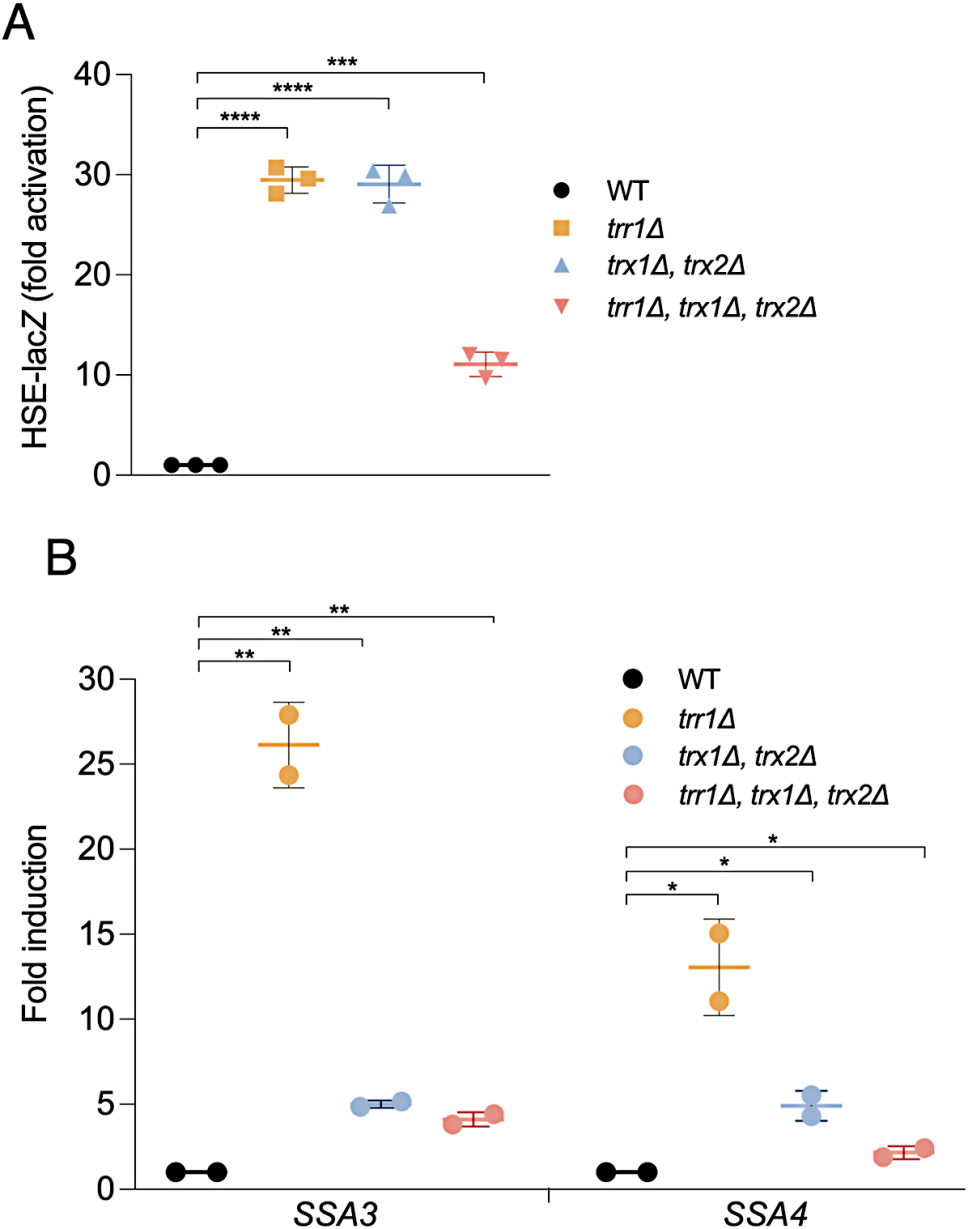
Cells deficient in the thioredoxin system exhibit constitutive activation of the HSR. (A) The indicated strains bearing the p*SSA3HSE-lacZ* reporter plasmid were grown to mid-logarithmic phase and β-galactosidase activity measured as described in the *Materials and Methods*. (B) The indicated strains were grown to midlogue phase, RNA extracted and levels of the *SSA3* and *SSA4* transcripts determined by qRT-PCR using the *TAF10* gene as a normalization control. Statistical significance between the indicated strains was determined using Welch’s unpaired *t* test (*p* = 0.05, *; *p* = 0.005, **; *p* = 0.0005, ***; *p* = 0.00005, ****). Values shown are the mean with SD and represent three (A) or two (B) biological replicates.

Based on previous work demonstrating that thiol-reactive compounds are potent activators of the HSR, we sought a genetic approach to alter redox balance in the absence of exogenous insults (Trott *et al.*, 2008; Wang *et al.*, 2012). To accomplish this, cells lacking the *TRR1* and/or redundant *TRX1* and *TRX2* genes were transformed with a β-galactosidase transcriptional reporter plasmid driven by a typical heat shock element-containing minimal promoter (HSE-*lacZ*). This reporter specifically monitors Hsf1 transcriptional activity and has been successfully used as a sensitive indicator of HSR status (Bonner *et al.*, 1994; Morano *et al.*, 1999). Reporter activity was strikingly elevated in *trr1∆*, *trx1∆ trx2∆*, and *trr1∆ trx1∆ trx2∆* cells as compared with wild type cells under nonstress conditions (Figure 1A). While the *trr1∆* and *trx1∆ trx2∆* strains exhibited nearly identical elevated levels of constitutive HSE-*lacZ* activity, the triple mutant displayed a lower, but still heightened, level of activation. To further explore the connections between cellular redox buffering systems and HSR regulation, we assessed HSE-*lacZ* in additional genetic backgrounds. Consistent with their redundant nature, cells lacking either the thioredoxin genes *TRX1* or *TRX2* exhibited no change in the HSR (Supplemental Figure S1). A similar outcome was observed in cells deficient in the mitochondrial thioredoxin reductase system (*trr2∆* and *trx3∆*; Trotter and Grant, 2005). The methionine sulfoxide reductase Mxr1 is required to reduce oxidized methionine residues (Kaya *et al.*, 2010). Deletion of *MXR1* likewise did not affect HSE-*lacZ* activity. We additionally tested the role of the glutathione system in HSR regulation. Loss of the glutathione reductase *GLR1* did not activate the HSR. Synthesis of glutathione requires the gene *GSH1*, encoding gamma glutamylcysteine synthetase, and *gsh1∆* mutants are inviable in the absence of exogenous reduced glutathione (Spector *et al.*, 2001). We, therefore, cultured *gsh1∆* cells in the presence of 1 mM glutathione, followed by washout and growth for an additional 6 h in the presence or absence of glutathione and observed no change in HSE-*lacZ* activity. We confirmed the reporter assay results by measuring endogenous transcript levels of the *SSA3* and *SSA4* Hsp70 genes via qRT-PCR (Figure 1B). Together, these data demonstrate that the cytoplasmic thioredoxin system is uniquely linked to Hsf1 transcriptional activation in *S. cerevisiae* cells grown under optimal, normoxic conditions.

### Thioredoxin system mutants hyperaccumulate the sequestrase Hsp42

**FIGURE 2:**
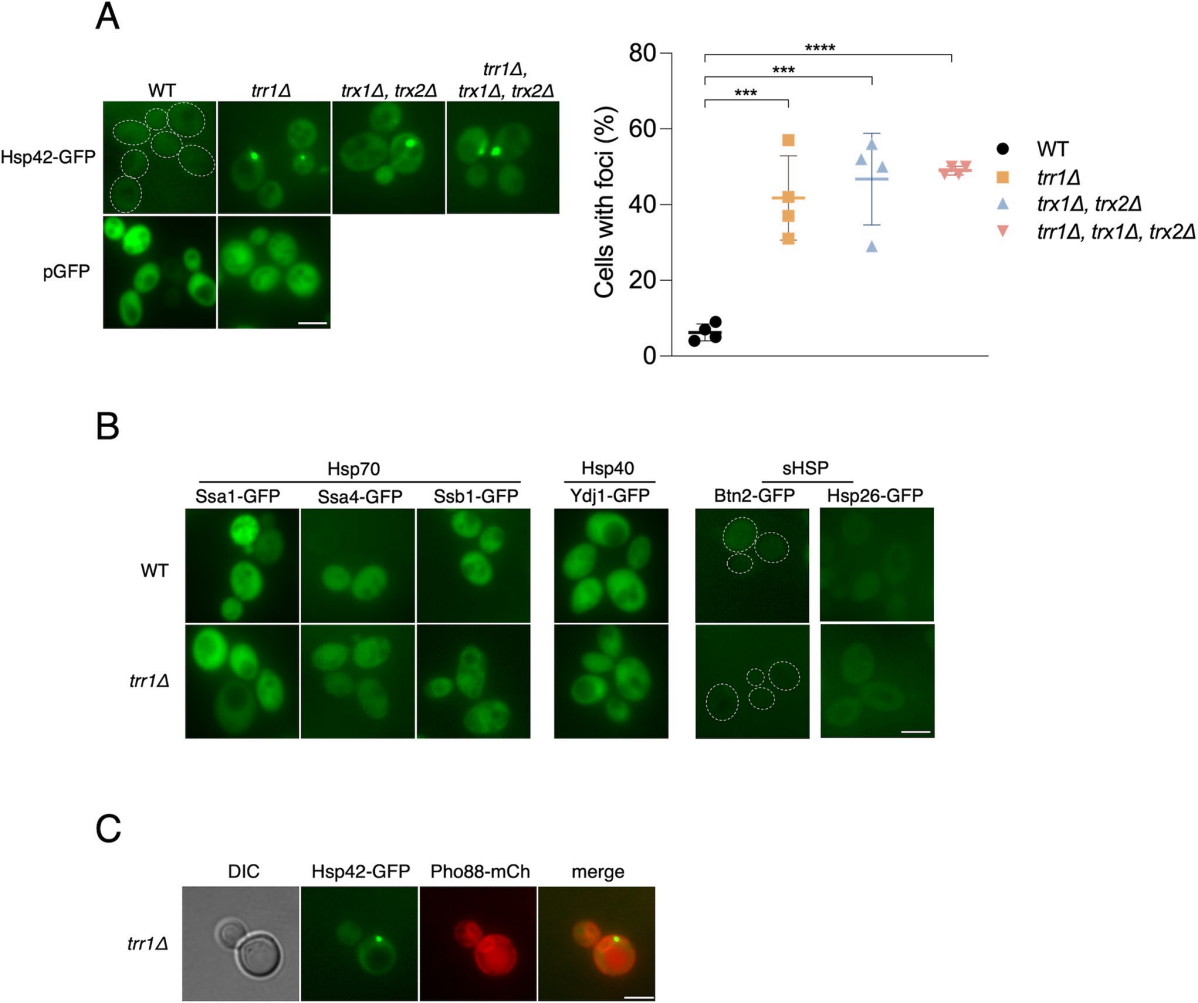
Thioredoxin system mutants hyperaccumulate the sequestrase Hsp42. (A) The indicated strains with GFP integrated in-frame with *HSP42*, or transformed with a plasmid expressing GFP alone, were grown to mid-logarithmic phase and imaged as described in *Materials and Methods*. The percentage of cells with foci was determined by counting at least 100 cells from multiple fields. (B) Wild type (WT) or *trr1∆* cells expressing GFP fusions to the indicated genes at the genomic locus were imaged in midlogue phase. (C) Strain *HSP42-GFP trr1∆ PHO88-mCherry* was grown to midlogue phase and imaged as described in *Materials and Methods*. All experiments included three (four for (A)) biological replicates and values are the mean with error bars representing SD. Statistical significance between the indicated strains was determined using Welch’s unpaired *t* test (*p* = 0.05, *; *p* = 0.005, **; *p* = 0.0005, ***; *p* = 0.00005, ****). Scale bar = 5 µm.

The observed chronic HSR activation in thioredoxin-mutant cells suggested that one or more aspects of general cellular proteostasis is negatively impacted in the absence of the thioredoxin system. Previous work has established that misfolded proteins are collected into transient deposition sites for storage and/or processing, which may include ubiquitination and eventual degradation by the proteasome (Miller *et al.*, 2015b). One of the first steps in this process for cytoplasmic proteins is the formation of Q-bodies (also called CytoQ) that typically include the sequestrase Hsp42, one or more Hsp70 proteins (Ssa1, Ssa2, Ssa3, and Ssa4) and their cofactors (Sse1/2, Ydj1, and Sis1), and the disaggregase Hsp104. Q-bodies containing misfolded proteins either resolve via refolding catalyzed by the abovementioned chaperones or coalesce into larger depots termed juxtanuclear (JUNQ) or intranuclear (INQ) quality control compartments (Kaganovich *et al.*, 2008; Miller *et al.*, 2015b; Hill *et al.*, 2017). We employed fluorescence microscopy to assess the status of multiple chaperone proteins using chromosomally encoded green fluorescent protein (GFP) fusions. Strikingly, while diffuse in wild type cells, Hsp42-GFP accumulated at a high frequency (40–60% of cells) in large, generally single foci in cells lacking cytoplasmic thioredoxin reductase, both cytoplasmic thioredoxins, or all three proteins (Figure 2A). Cells expressing GFP alone did not exhibit concentration of fluorescence signal. Because we observed no significant differences between the *trr1∆* and *trx1∆ trx2∆* strain backgrounds in our studies, all further experiments were carried out using the former deletion strain to inactivate the thioredoxin system. GFP fusions to the Hsp70s Ssa1, Ssa4, Ssb1, and the Hsp40 chaperone Ydj1 also remained diffuse in *trr1∆* cells (Figure 2B). The chaperones Btn2 and Hsp26, as divergent members of the sHSP family, contain loosely conserved α-crystallin chaperone domains as well as divergent amino- and carboxyl-terminal regions that dictate differential behavior with respect to substrate recruitment (Supplemental Figure S2A; Mogk *et al.*, 2019). Additionally, Btn2 possesses protein sequestrase activity similar to Hsp42 (Malinovska *et al.*, 2012; Miller *et al.*, 2015a). However, GFP fusions to both proteins remained diffusely localized in *trr1∆* cells, with low signal intensity in our experiments (Figure 2B). The cellular levels of Btn2 and Hsp26 are quite low in unstressed wild type cells, but increased in *trr1∆* cells, as are levels of Hsp42 as assessed by immunoblot (Supplemental Figure S2B). These results are consistent with all three being under the transcriptional control of Hsf1 and suggest that the presence of the large Hsp42 foci is not due to overexpression of the chaperone (Solís *et al.*, 2016). To confirm that the altered localization pattern of Hsp42 was due to the enzymatic activity of Trr1 and its role in maintaining proper redox balance in the cytoplasm, we generated an Hsp42-GFP strain wherein the catalytic and resolving cysteine residues of Trr1 required for the disulfide exchange reaction were substituted with serines (Chae *et al.*, 1994). Cells expressing the *TRR1-C142S-C145S* allele exhibited the same high level of Hsp42-GFP focus formation as *trr1∆* cells bearing an empty vector, as well as the drastic slow-growth phenotype of cells lacking thioredoxin reductase activity (Supplemental Figure S3, A and B; Trotter *et al.*, 2002; Kritsiligkou *et al.*, 2018).

In addition to the JUNQ and INQ compartments, a third depot termed the IPOD has been described in yeast that typically includes amyloid-forming proteins as well as Hsp42 and other chaperones (Kaganovich *et al.*, 2008; Rothe *et al.*, 2018). While JUNQ and INQ are perinuclear and intranuclear, respectively, IPOD is typically perivacuolar or randomly localized within the cytoplasm. To assess where Hsp42-GFP was localizing in *trr1∆* cells, we constructed a strain expressing an mCherry-tagged allele of the ER membrane protein Pho88 that also outlines the nuclear membrane (D’Urso *et al.*, 2016). The Hsp42-GFP signal was generally observed to be immediately adjacent to the cytoplasmic face of the nuclear membrane as judged by comparison to the Pho88-mCh signal, consistent with accumulation/persistence of a JUNQ-like compartment in *trr1∆* cells (Figure 2C).

### Chaperone and substrate dynamics in *trr1*∆ cells

**FIGURE 3:**
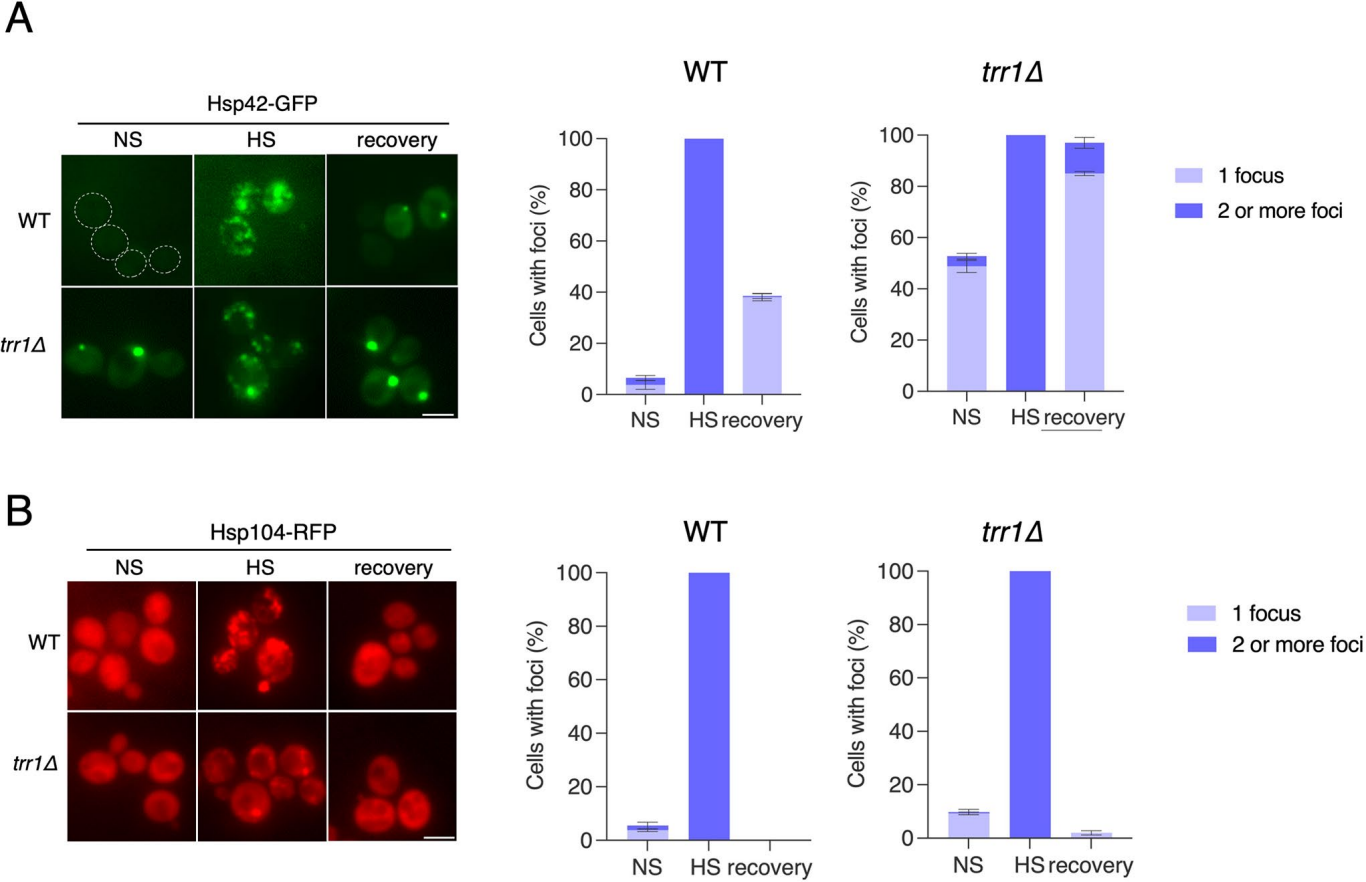
Chaperone and substrate dynamics in *trr1∆* cells. The indicated strains expressing Hsp42-GFP (A) or Hsp104-RFP (B) from the chromosomal locus were grown to mid-logarithmic phase at 30°C (NS), shifted to 39°C for 20 min (HS), and allowed to recover for 5 h at 30°C (recovery). The percentage of cells with one or two or more foci was determined by counting at least 100 cells from multiple fields. All experiments included three biological replicates and values are the mean with error bars representing SD. Scale bar = 5 µm.

To further explore the nature of the large Hsp42 foci in *trr1∆* cells, we assessed chaperone localization in response to protein misfolding stress. Wild type and *trr1∆* cells bearing either Hsp42-GFP or a red fluorescent protein (RFP) fusion to the disaggregase Hsp104 (Hsp104-RFP) were grown at the nonstress temperature of 30°C and shifted or not to 39°C (HS) for 20 min. Heat-shocked cells were then returned to 30°C for a 5-h recovery period. As expected, wild type cells exhibited diffuse fluorescence in control conditions, and formed multiple small foci in nearly all cells (CytoQ) during heat shock (Figure 3A). These CytoQ bodies were largely resolved into a single focus in ∼40% of cells during the recovery period. In *trr1∆* cells, the same CytoQ dynamics were observed, while the single large foci remained present during all phases of the experiment. Interestingly, the persistence of large, single foci, presumably exaggerated JUNQ compartments, was greater in *trr1∆* cells than in wild type cells (over 80% vs. ∼40%) at the end of the recovery period, suggesting that the resolved CytoQ bodies were “added” to the redox-dependent body. This possibility is further supported by the fact that we did not observe two independent large foci in Hsp42-GFP *trr1∆* cells after the recovery phase. We performed the same experiment using Hsp104-RFP-containing cells and noted that while both wild type and *trr1∆* strains accumulated CytoQ during the heat shock, in neither strain were persistent large foci observed under control or recovery conditions (Figure 3B). These findings establish that Hsp42, like Hsp104, is rapidly recruited to CytoQ structures under protein misfolding stress; however, Hsp42 fails to disengage during the recovery phase in contrast to Hsp104. Moreover, in *TRR1*-deficient cells, CytoQ dynamics proceed normally with the addition of persistent JUNQ compartments containing Hsp42.

### Terminally misfolded proteins accumulate in Hsp42-GFP foci in *trr1*∆ cells

**FIGURE 4:**
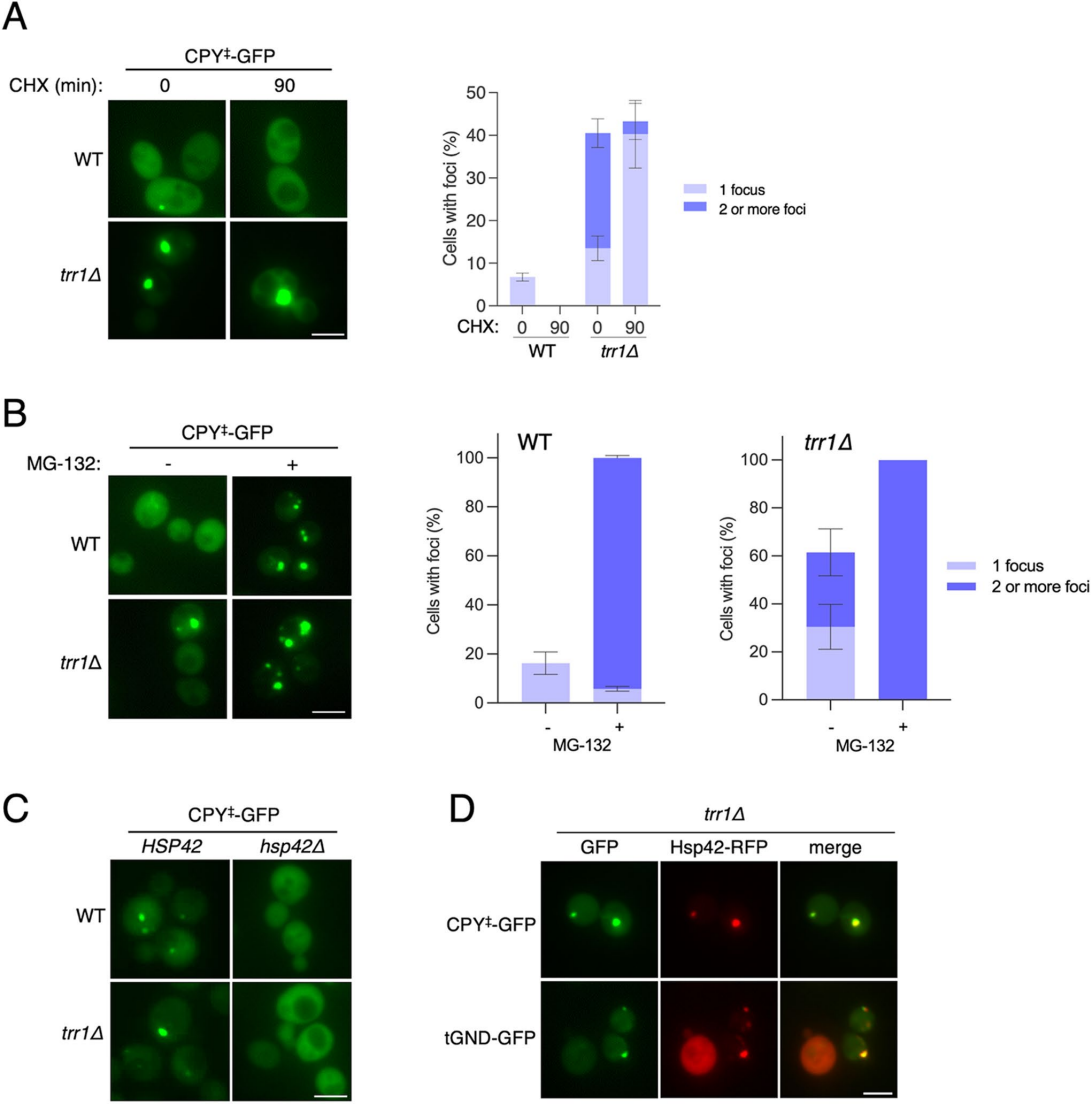
Terminally misfolded proteins accumulate in Hsp42-GFP foci in *trr1∆* cells. (A) The indicated strains expressing the CPY^‡^-GFP misfolded protein fusion from the chromosome were grown at 30°C and imaged immediately before (0) or 90 min after (90) treatment with CHX as described in *Materials and Methods*. The percentage of cells with one or two or more foci was determined by counting at least 100 cells from multiple fields. (B) The indicated strains (additionally *pdr5∆*) were grown at 30°C in the absence (–) or presence (+) of MG-132 for 2 h. The percentage of cells with one or two or more foci was determined by counting at least 100 cells from multiple fields. (C) The indicated strains were grown to mid-logarithmic phase at 30°C and imaged. (D) Strains expressing Hsp42-RFP as well as either CPY^‡^-GFP or tGND-GFP were grown to midlogue phase and imaged in both the red and green channels. All experiments included three biological replicates and values are the mean with error bars representing SD. Scale bar = 5 µm.

The Hsp42 sequestrase forms multimeric scaffolded structures with misfolded proteins that both prevents their aggregation and facilitates extraction and refolding as well as degradation (Mogk and Bukau, 2017). The persistence of JUNQ compartments containing Hsp42-GFP in cells defective in the thioredoxin system suggested that misfolded proteins might also accumulate within these structures. To test this hypothesis, we expressed the terminally misfolded model protein CPY^‡^-GFP in wild type and *trr1∆* cells and monitored localization dynamics in the presence or absence of the translation inhibitor cycloheximide (CHX; Heck *et al.*, 2010). CPY^‡^-GFP typically forms small CytoQ bodies that are resolved over time via degradation by the proteasome (Figure 4A). In contrast, in *trr1∆* cells, CPY^‡^-GFP accumulated in one to two large foci, and, after 90 min of CHX chase, the majority of cells exhibited a single very large focus reminiscent of the Hsp42-GFP/JUNQ compartment. These results suggest that the CPY^‡^-GFP within the CytoQ bodies failed to be extracted and degraded in *trr1∆* cells and instead hyperaccumulated into an exaggerated JUNQ compartment. Indeed, when the same experiment was performed using the proteasome inhibitor MG-132 instead of CHX, both wild type and *trr1∆* cells displayed similar accumulation of two or more exaggerated foci, consistent with a degradation block (Figure 4B). Accumulation of CPY^‡^-GFP into foci of any size was entirely dependent on Hsp42 as previously reported, as no foci were detected in both *TRR1 hsp42∆* and *trr1∆ hsp42∆* cells (Figure 4C; Specht *et al.*, 2011; Saarikangas and Barral, 2015). We also examined whether Hsp104 colocalized with CPY^‡^-GFP in MG132-treated cells and found that the disaggregase remained diffuse regardless of *TRR1* status, consistent with our data and others showing that Hsp104 transiently accumulates in CytoQ bodies but is not a permanent resident (Supplemental Figure S6). Finally, to verify that the Hsp42-GFP and misfolded protein foci accumulating in *trr1∆* cells are one and the same, we generated an Hsp42-RFP fusion protein and coexpressed it with either CPY^‡^-GFP or another permanently misfolded substrate, the truncated Gnd1 protein (tGND-GFP; Heck *et al.*, 2010). In both cases, the foci completely overlapped. Together, these results demonstrate that the JUNQ compartments persisting in *trr1∆* cells contain both the sequestrase Hsp42 as well as misfolded proteins. These proteins are likely associated with each other in a manner that precludes degradation in the case of the two-model proteins and refolding in the case of endogenous substrates normally processed through transient CytoQ assemblies.

One explanation for the persistent JUNQ structures could be excess production of misfolded proteins in *trr1∆* cells; that is, an overall increase in protein misfolding due to an altered redox environment. To test this possibility, we expressed the thermally labile protein firefly luciferase (FFL) as a GFP fusion protein in wild type and *trr1∆* cells (Abrams and Morano, 2013). FFL-GFP misfolds under proteotoxic stress conditions and localizes to transient CytoQ bodies (Tkach and Glover, 2008; Escusa-Toret *et al.*, 2013; Miller *et al.*, 2015a). We, therefore, heat-shocked FFL-GFP-bearing cells, followed by return to normal temperature in the presence of CHX, and assessed both luciferase enzymatic activity and focus formation. FFL-GFP recovered ∼50% of initial activity in wild type cells over 90 min, with a moderately faster and higher rate of recovery in *trr1∆* cells (Supplemental Figure S4A). Consistent with these results, CytoQ punctae formed in both strains during heat shock and partially resolved by 90 min to a similar degree as assessed by fluorescence microscopy (Supplemental Figure S4B). While not an exhaustive analysis, these data suggest that global protein refolding is not grossly impaired in *trr1∆* cells.

An alternative explanation for the persistence of both misfolded protein like CPY^‡^-GFP and tGND-GFP in Hsp42-containing JUNQ compartments might be failure to properly target these substrates for degradation through the UPS (Baker and Bernardini, 2021). We assessed this possibility in three different ways. The addition of polyubiquitin chains to cellular misfolded proteins can be detected by immunoblot using antiubiquitin antibodies if downstream proteasome degradation is blocked. We, therefore, treated wild type and *trr1∆* cells with either MG-132, CHX or both compounds and prepared whole cell extracts for immunoblot. A characteristic smear of ubiquitin-positive proteins was observed in untreated cells whose intensity decreased with CHX (due to fewer nascent chains being produced) and enhanced with both CHX and MG-132 (due to proteasome inhibition) in both wild type and *trr1∆* cells, suggesting that the ubiquitination phase of protein quality control was unaffected by redox imbalance (Supplemental Figure S5A). To ask whether proteasome activity itself might be abrogated in *trr1∆* cells, leading to accumulation of misfolded proteins, we prepared native cell extracts and utilized a commercial proteasome enzymatic activity assay. 20S chymotrypsin-like proteasome activity was measured and validated by comparison with measurements taken in the presence of MG-132. Surprisingly, 20S proteasome activity was approximately threefold higher in *trr1∆* cells as compared with wild type, possibly due to enhanced proteasome gene expression driven by the *HSF1-RPN4* circuit (Supplemental Figure S5B; Hahn *et al.*, 2006). Finally, to rule out alteration specifically in 26S proteasome function of *trr1*∆ cells, we utilized N-end rule degron substrates including a destabilizing N degron Ub-R-KK-YFP-Su9 (Ub-R) and a stabilizing N degron Ub-V-KK-YFP-Su9 (Ub-V). The Ub-R (N-terminal arginine) is subject to rapid ubiquitination and subsequent degradation while the Ub-V (valine substitution) is not (Yu *et al.*, 2016). In the absence of MG-132, the Ub-R construct was present at very low levels and further destabilized in *trr1*∆ cells, while Ub-V levels were abundant in both strains, indicating no reduction in 26S proteasome activity in *trr1*∆ cells. MG-132 dramatically stabilized the Ub-R protein fusions, confirming proteasomal targeting (Supplemental Figure S5C). Together, these results demonstrate that overall function of the UPS is intact, if not elevated, in cells lacking thioredoxin reductase activity.

### Cells deficient in both thioredoxin reductase activity and sequestrase function experience synthetic slow growth and hypersensitivity to oxidative stress

**FIGURE 5:**
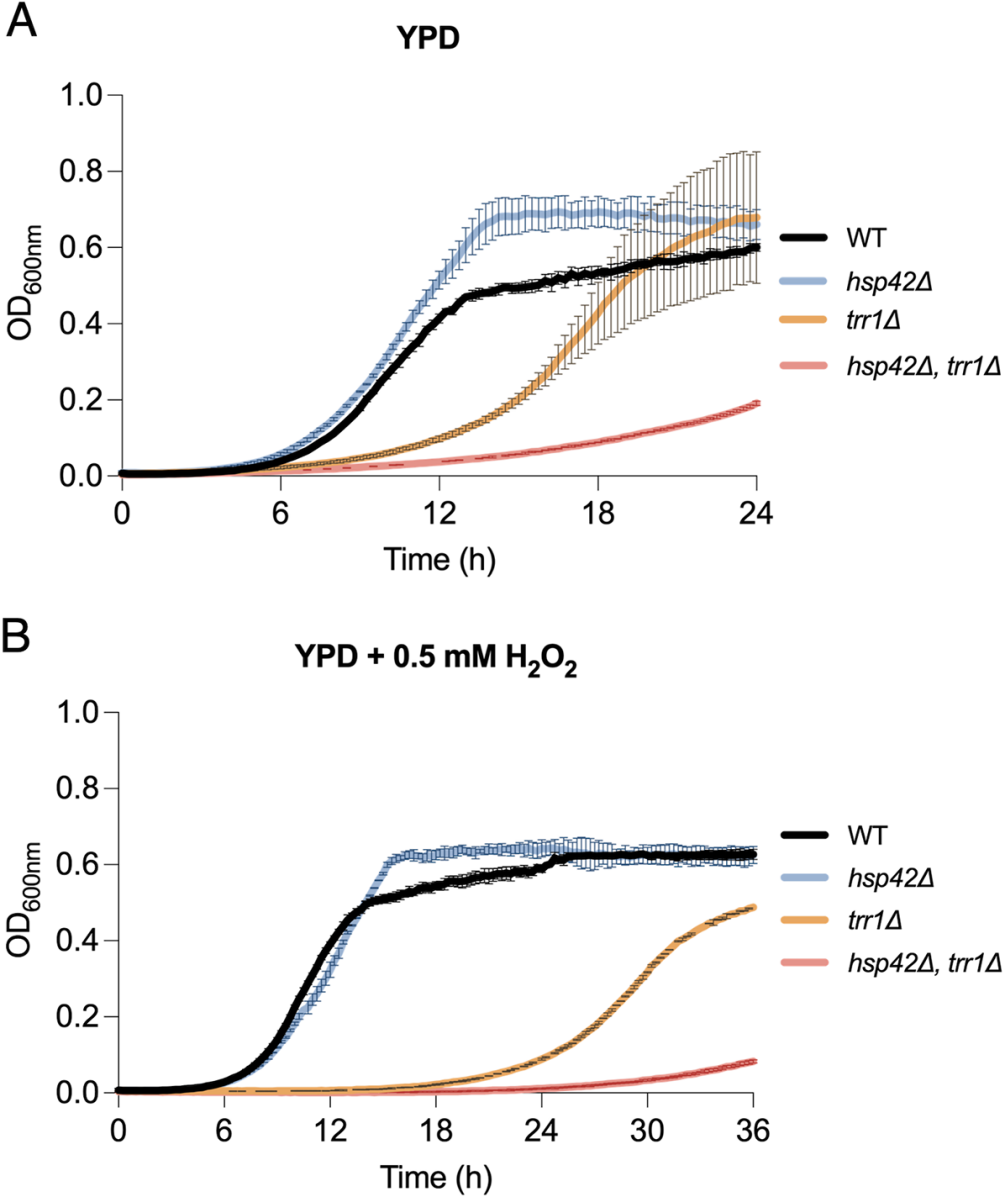
Cells deficient in both thioredoxin reductase activity and sequestrase function experience synthetic slow growth and hypersensitivity to oxidative stress. (A) The indicated strains were inoculated at an initial OD_600_ = 0.01 in a sterile 96-well plate and grown with shaking at 30°C for 24 h with density measurements taken every 10 min. The average of three biological replicate growth curves are shown with SD. (B) Same as (A), but cultures were additionally grown in the presence of 0.5 mM H_2_O_2_ for 36 h. Plotted values represent the mean of three biological replicates and error bars the SD.

Our experiments demonstrated that *trr1∆* cells accumulated both Hsp42 and misfolded proteins in an exaggerated JUNQ compartment that failed to resolve despite an intact UPS. Notwithstanding a near-absolute requirement for Hsp42 to form CytoQ or JUNQ structures in yeast cells, *hsp42∆* cells exhibit essentially no phenotypes unless cells are severely compromised for protein quality control through the Hsp70 chaperone network (Ho *et al.*, 2019). However, because our findings suggested that Hsp42 may be a critical chaperone in cells that lack the ability to maintain a reduced cytoplasm, we generated an *hsp42∆ trr1∆* strain and compared its growth to wild type and single-mutant strains. As previously reported, *trr1∆* cells exhibited a slow growth phenotype under normal conditions, while *hsp42∆* cells grew indistinguishably from wild type. In contrast, *hsp42∆ trr1∆* cells exhibited profoundly slow growth (Figure 5A). To ask whether exogenous oxidative stress may exacerbate these effects, the same four strains were cultured in the presence of 0.5 mM H_2_O_2_. A similar pattern was observed, with more intense growth retardation of both *trr1∆* and *hsp42∆ trr1∆* cells, the latter of which were nearly inviable. Together, these data highlight the interconnectedness of sequestrase and thioredoxin reductase functions and to our knowledge represent a rare example of synthetic growth defects resulting from genetic disruption of the *HSP42* locus in yeast.

### Hsp42 dynamics in glucose-starved and chronologically aging cells phenocopy *trr1*∆ cells

**FIGURE 6:**
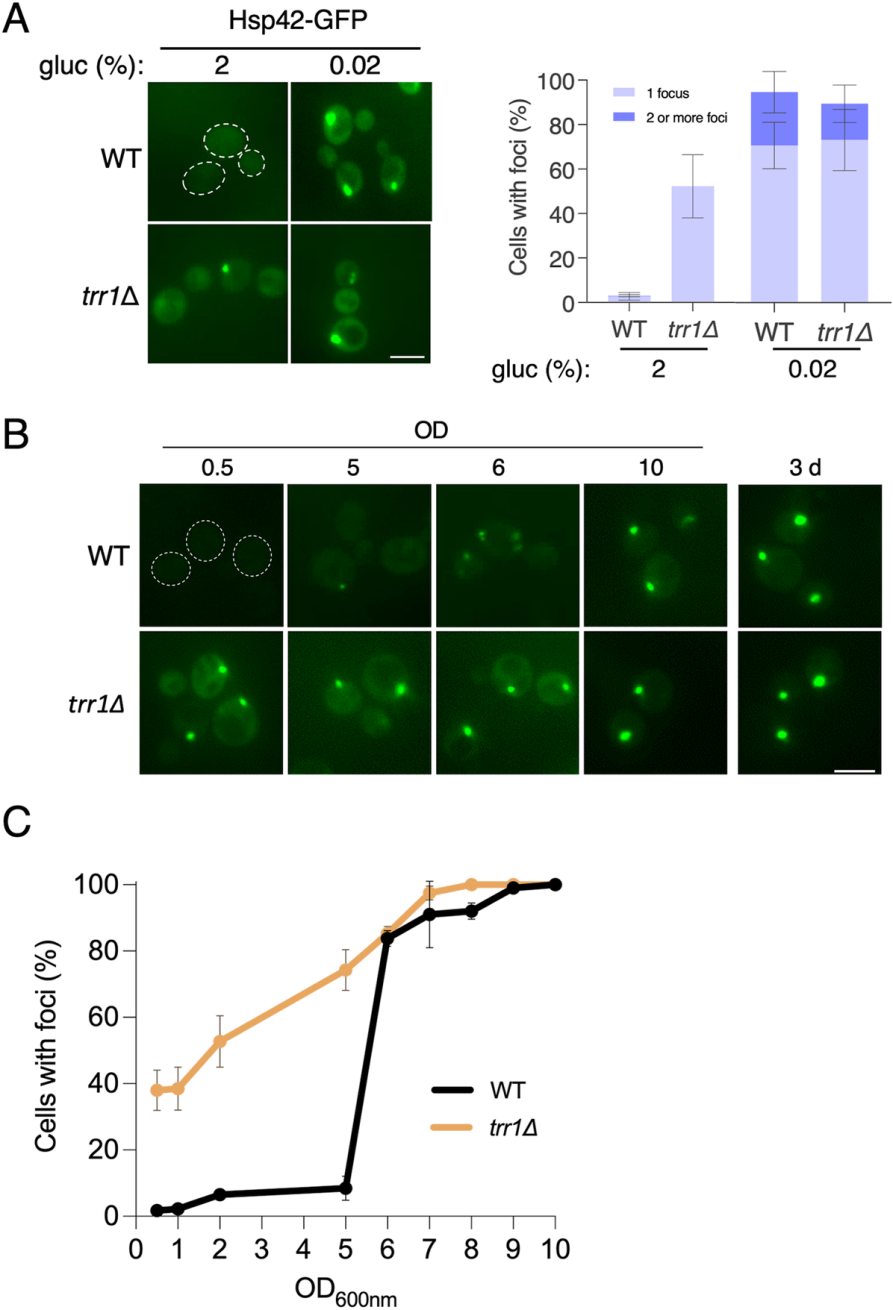
Hsp42 localization in glucose-starved and chronologically aging cells is phenocopied in *trr1∆* cells. (A) The indicated strains were grown at 30°C to mid-logarithmic phase and then either maintained in standard YPD medium or shifted to low glucose medium (YP + 0.02% glucose) for 90 min. The percentage of cells with one or two or more foci was determined by counting at least 100 cells from multiple fields. (B) The same strains as in (A) were grown at 30°C and culture aliquots removed at the indicated optical densities (OD) or after 3 d of continuous growth. (C) The percentage of cells with foci was determined by counting at least 100 cells from multiple fields. All experiments included three biological replicates and values are the mean with error bars representing SD. Scale bar = 5 µm.

Yeast growth in a fixed culture over time has been interpreted as a microbial model of eukaryotic aging; cells initially exhibit robust growth in a nutrient-rich environment that slows as key nutrients, including glucose, are exhausted (Herman, 2002). The same metabolic state can be achieved by shifting logarithmically growing, glucose-rich (2%) cultures to a minimal glucose concentration (0.02%), bringing about acute glucose starvation. Hsp42 has been previously shown to accumulate in so-called “Hsp42 bodies” in yeast cultures during stationary phase or in response to glucose starvation that bear a strong resemblance to the enhanced JUNQ structures observed in our experiments (Narayanaswamy *et al.*, 2009; Liu *et al.*, 2012; O’Connell *et al.*, 2014; Lee *et al.*, 2016). We, therefore, sought to better understand possible similarities between all these observations. Wild type and *trr1∆* cells were grown in rich glucose media and then shifted to low glucose conditions and Hsp42-GFP focus accumulation assessed. While *trr1∆* cells exhibited a high percentage of single-focus formation in both growth environments, wild type cells only accumulated an Hsp42-GFP focus under starvation conditions (Figure 6A). In fact, although the percentage of single foci increased in the *trr1∆* strain after shift to low glucose (50 to ∼75%), the total percentage was nearly identical in both strains. We interpret this result to mean that *trr1∆* cells largely phenocopy the glucose-starved scenario in wild type cells and that acute starvation is minimally additive with genetically induced redox imbalance. To ask whether the same concept held true for batch culture aging, wild type and *trr1∆* cells were grown in parallel cultures and images taken of Hsp42-GFP localization at defined culture densities. Similar to the acute glucose shift experiment, *trr1∆* cells exhibited a high frequency of focus formation during exponential phase that further increased in the aging culture (Figure 6, B and C). In contrast, no foci were observed in wild type cells until a threshold at approximately OD_600_ = 5, after which nearly all cells contained one or two large JUNQ foci. At 3 d of continuous culture, Hsp42-GFP localization patterns in the two strains were virtually indistinguishable, suggesting that these phenomena may be similar in nature if not etiology, hinting at a potential common origin that connects redox maintenance with cellular energy levels.

## DISCUSSION

Our work in redox-imbalanced cells has revealed a profound dysregulation of not only the protein misfolding-induced HSR, but also spatial quality control, manifest as specific and persistent hyperaccumulation of the cytosolic sequestrase Hsp42. How does loss of either thioredoxin reductase or the redundant cytosolic thioredoxins Trx1/2 lead to Hsf1 activation? Recent work has conclusively demonstrated that the Hsp70 chaperone proteins Ssa1/2 restrain Hsf1 in an inactive state through direct physical interaction at sites within the disordered amino- or carboxyl-terminal domains of the transcription factor (Zheng *et al.*, 2016; Peffer *et al.*, 2019; Chowdhary *et al.*, 2022). The Ssa1-Hsf1 complex exhibits decreased DNA binding and nuclear condensate clustering, resulting in diminished RNA polymerase II recruitment at target gene promoters. Unfolded or misfolded polypeptides displaying exposed Hsp70 binding motifs compete with Hsf1 for Ssa1 binding, leading to HSR activation (Zheng *et al.*, 2016). Protein misfolding due to redox imbalance could arise from defects in translation or multimerization/assembly of proteins containing one or more redox-active cysteine or methionine residues. However, we demonstrated that the heat-labile enzyme luciferase folds and acquires enzymatic activity in *trr1∆* cells, arguing against a general protein folding defect. Alternatively, abridged function of the ubiquitin-proteasome pathway could generate a pool of misfolded proteins that compete for Hsf1-bound Ssa1 by blocking degradation, but our observation that bulk ubiquitination is unaffected in *trr1∆* cells is inconsistent with this idea. We favor a model wherein impaired mobilization from CytoQ/JUNQ compartments results in a pool of accumulated misfolded proteins. This last scenario comports both with the persistent Hsp42 foci observed in *trr1∆* cells and our findings that the unfoldable substrates CPY^‡^-GFP and tGnd1-GFP colocalized with these structures. Finally, it is possible that two independent mechanisms may be in play, as we have shown that reactive cysteines 264 and 303 in Ssa1 are subject to oxidative modification that inhibits general chaperone activity and releases Hsf1 (Santiago and Morano, 2022). Indeed, a pool of partially oxidized Ssa1 may exist in *trr1∆* cells that both contributes to improper spatial quality control and improperly restrains Hsf1.

Notably, HSR activation was observed exclusively in mutant strains deficient in the cytosolic thioredoxin system. Elimination of other redox pathways, including the mitochondrial thioredoxin system, the cytosolic/vacuolar glutathione system, or the Mxr1 methionine-S-oxide reductase, did not result in elevated Hsf1 transcriptional activity. While the glutathione pathway plays a major role in redox balance in most eukaryotic cells, including humans, its importance in *S. cerevisiae* is focused on detoxification, antioxidant function, amino acid biosynthesis and iron-sulfur cluster biogenesis, and may serve a subordinate role to the thioredoxin system. Interestingly, it was previously demonstrated that the endoplasmic reticulum-localized unfolded protein response pathway (UPR) is constitutively activated in *trr1∆* cells, likely due to the shift toward oxidized thioredoxins in the absence of thioredoxin reductase, disallowing proper disulfide bond rearrangement by the protein disulfide isomerase Pdi1 (Kritsiligkou *et al.*, 2018). In a similar manner, cytosolic protein cysteines could become oxidized in an environment lacking transferable reducing equivalents normally supplied by thioredoxins and the peroxiredoxin family. In support of this model, the peroxiredoxin Tsa1 localizes with oxidatively damaged, misfolded proteins and converts to a functional chaperone upon cysteine oxidation (Hanzén *et al.*, 2016). We note that Hsp42 also contains a single cysteine residue located at position 127. This residue lies within the amino-terminal domain required for localization to peripheral structures including CytoQ and JUNQ, raising the possibility that C127 oxidation could impact Hsp42 localization dynamics (Specht *et al.*, 2011). Btn2 contains five cysteine residues, but like Hsp42 C127, it is unknown whether they are redox-active (Saccharomyces Genome Database | SGD). A deeper dive into potential redox dynamics of the sequestrases is therefore warranted. Importantly, we are not the first to observe dysregulation of the HSR in *trr1∆* cells. MacDiarmid and coworkers identified a *TRR1*-inactivating genetic suppressor of *tsa1∆* growth defects in cells deficient in zinc, and further showed that *trr1∆* cells exhibited constitutive activation of the HSR that was exacerbated by zinc limitation (Macdiarmid *et al.*, 2013). We have significantly extended these findings by identifying additional, previously unknown defects in spatial quality control in the absence of thioredoxin reductase in cells with no zinc challenge.

What is the nature of the exaggerated Hsp42-containing JUNQ compartment in *trr1∆* cells? We found that other chaperones typically associated with CytoQ bodies and the JUNQ, such as Hsp40 (Ydj1), Hsp70 (Ssa1, Ssa4, and Ssb1) and the disaggregase Hsp104, did not accumulate along with Hsp42 and misfolded protein. These chaperones transiently localize to CytoQ/JUNQ and participate in the extraction of polypeptides for further processing – refolding or proteasomal degradation. In fact, we found that Hsp104 and Hsp42 appropriately localized to CytoQ structures during acute heat shock. Hsp104 then appeared to return to a diffuse state while Hsp42 did not. Because large Hsp42-containing foci both preceded the heat shock experiment and persisted during recovery, we cannot distinguish whether the transient CytoQ-localized Hsp42 resolved like Hsp104 or became trapped within the accumulating JUNQ compartment. However, the impact of redox imbalance on spatial quality control seems specific to Hsp42 and likely associated misfolded proteins, rather than a general defect in protein processing. This point is underscored by our observation that the related sequestrase Btn2, associated with the INQ compartment, did not accumulate in detectable foci in *trr1∆* cells, nor did the holdase Hsp26. Further work will be required to identify a mechanistic explanation for aberrant Hsp42 cellular dynamics. However, the importance of Hsp42 in cells experiencing redox imbalance through loss of Trr1 is underscored by the dramatic synergistic growth phenotype observed in *trr1∆ hsp42∆* cells. This growth defect is even more striking when considered in light of the fact that cells lacking *HSP42* exhibit little to no demonstrable phenotypes of any kind unless the Hsp70 system is compromised (Haslbeck *et al.*, 2004; Ho *et al.*, 2019). Clearly, Hsp42 is required to support proliferation of *trr1∆* cells which already experience profound growth retardation linked to ROS production in the ER. Our observation that *trr1∆ hsp42∆* growth rates are even further compromised during oxidative stress is consistent with a model wherein cytosolic sequestrase activity is critical in a challenging redox environment. This hypothesis is further supported by recent work demonstrating that Hsp42, and to a lesser extent, Btn2, are required for tolerance to exogenous acute oxidative stress (Carter *et al.*, 2023).

A notable feature of this work is the connection between our observations and previous studies identifying Hsp42 bodies forming in response to nutrient starvation and chronological aging. Indeed, it is difficult to distinguish Hsp42-GFP localization patterns under the various conditions. Our chronological aging experiment demonstrates that the exaggerated single foci that chronically persist in *trr1∆* cells closely resemble the same structures that begin to accumulate in postlogarithmic phase growth, which in turn resemble cells experiencing acute glucose starvation (0.02% glucose). While it is tempting to speculate that the observed nutrient starvation effects are, therefore, due to redox imbalance, we also note that the percentage of the *trr1∆* cell population with Hsp42 foci increased during the aging experiment as well as upon shift to limiting glucose, suggestive of a partially additive relationship. Hsp42 localization patterns were not appreciably different in wild type or *trr1∆* cells at 0.02% glucose, further suggesting that carbon source starvation (and hence energy production) is a more severe proteotoxic insult with respect to Hsp42/JUNQ dynamics than redox imbalance. Many questions remain to be answered regarding the links between redox homeostasis, energy state, and spatial quality control. Why is Hsp42 localization (JUNQ) so drastically altered with no detectable change in Btn2 dynamics (INQ) given the interconnectedness of the two compartments (Sontag *et al.*, 2023)? What proteins constitute the exaggerated JUNQ compartment present in *trr1∆* and aging/starved cells, and are they similar among the different conditions? Finally, we seek to understand the cytoprotective role that Hsp42 appears to play in redox-challenged cells, presumably via sequestration of potentially toxic misfolded proteins.

## MATERIALS AND METHODS

Request a protocol through *Bio-protocol*.

### Strains and plasmids

All strains used in this study are isogenic to BY4741 and are listed in Table 1. Multiple GFP fusion strains were from the Thermo Fisher Scientific GFP Collection (Huh *et al.*, 2003). Multiple gene deletion strains are from the Yeast Knockout Collection (Winzeler *et al.*, 1999). Construction of knockout strains was done by generating PCR amplicons containing one of the markers *G418^R^*, *LEU2*, or *URA3,* flanked by upstream and downstream noncoding regions of the knockout target gene. Alternatively, we employed the seamless deletion method to recycle the *URA3* gene and generate *trr1*Δ seamless strains, denoted as trr1∆* in Table 1 (Horecka and Davis, 2014). For expression of *TRR1* in *trr1*Δ strains, the genomic region of BY4741 containing the *TRR1* coding sequence and 1 kb upstream and 1 kb downstream was amplified and cloned into the pRS415 plasmid (Table 1) using introduced *Spe*I and *Xba*I restriction sites. The same plasmid construct was used for the generation of *TRR1-C142S-C145S*, where the codons encoding the two cysteines in the catalytic region of Trr1 (C142 and C145) were changed to encode serine using overlap PCR. The GFP-expressing control plasmid pRS415-GPD-GFP was constructed by amplifying the GFP coding sequence from the genomic DNA of the Hsp42-GFP strain and cloned into pRS415-GPD using introduced *Spe*I and *Xho*I restriction sites. All plasmid constructions and genomic integration cassettes were verified by sequencing before yeast transformation. Yeast transformation was performed using the rapid yeast transformation protocol (Gietz *et al.*, 1992). Plasmids pRH2081 (*TDH3-CPY^‡^-GFP*), pRH2476 (*TDH3-tGND-GFP*), and pRS303-*PHO88-mCherry* were linearized as previously described before integrative transformation (Heck *et al.*, 2010; D’Urso *et al.*, 2016).

**TABLE 1:** Yeast strains and plasmids.

Strains	Description	Reference
BY4741	*MATα his3∆1 leu2∆0 met15∆0 ura3∆0*	Laboratory stock
*trr1∆*	BY4741 trr1∆*	This study
*trx1∆*	BY4741 *trx1∆*::*G418^R^*	(Allan *et al.*, 2016)
*trx2∆*	BY4741 *trx2*::*HIS3*	(Allan *et al.*, 2016)
*trx1∆ trx2∆*	BY4741 *trx1∆*::*G418^R^ trx2*::*HIS3*	(Allan *et al.*, 2016)
*trx3∆*	BY4741 *trx3∆*::*G418^R^*	Yeast Knockout Collection
*trr2∆*	BY4741 *trr2∆*::*G418^R^*	Yeast Knockout Collection
*mxr1∆*	BY4741 *mxr1∆*::*G418^R^*	Yeast Knockout collection
*glr1∆*	BY4741 *glr1∆*::*G418^R^*	Yeast Knockout collection
*gsh1∆*	BY4742 *gsh1∆*::*G418^R^*	(Spector *et al.*, 2001)
*pdr5∆*	BY4741 *pdr5∆*::*G418^R^*	Yeast Knockout Collection
*pdr5∆ trr1∆*	BY4741 *pdr5∆*::G418^R^ trr1∆*	This study
*hsp42∆*	BY4741 *hsp42∆*::*G418^R^*	Yeast Knockout collection
*hsp42∆ trr1∆*	*hsp42∆ trr1∆*::*URA3*	This study
Hsp42-GFP	BY4741 *HSP42-GFP*::*HIS3*	Thermo Fisher Scientific
Hsp42-GFP *trr1∆*	Hsp42-GFP trr1∆*	This study
Hsp42-GFP *trx1∆ trx2∆*	Hsp42-GFP *trx1∆*::*G418^R^ trx2∆*::*LEU2*	This study
Hsp42-GFP *trx1∆ trx2∆ trr1∆*	Hsp42-GFP *trx1∆*::G418^R^*trx2∆*::LEU2 trr1∆*	This study
Hsp104-GFP	BY4741 *HSP104-GFP*::*HIS3*	Thermo Fisher Scientific
Hsp104-GFP *trr1∆*	Hsp104-GFP *trr1∆*::*URA3*	This study
Hsp104-GFP *trx1∆ trx2∆*	Hsp104-GFP *trx1∆*::*G418^R^ trx2∆*::*LEU2*	This study
Hsp104-GFP *trx1∆ trx2∆ trr1∆*	Hsp104-GFP *trx1∆*::*G418^R^ trx2∆*::*LEU2 trr1∆*::*URA3*	This study
Hsp104-RFP	*HSP104-yEmRFP*::*G418^R^*	Laboratory stock
Hsp104-RFP *trr1∆*	Hsp104-RFP *trr1∆*::*URA3*	This study
Hsp26-GFP	BY4741 *HSP26-GFP*::*HIS3*	Thermo Fisher Scientific
Hsp26-GFP *trr1∆*	Hsp26-GFP *trr1∆*::*URA3*	This study
Btn2-GFP	BY4741 *BTN2-GFP*::*HIS3*	Thermo Fisher Scientific
Btn2-GFP *trr1∆*	Btn2-GFP *trr1∆*::*URA3*	This study
Ssa1-GFP	BY4741 *SSA1-GFP*::*HIS3*	Thermo Fisher Scientific
Ssa1-GFP *trr1∆*	Ssa1-GFP *trr1∆*::*URA3*	This study
Ssa4-GFP	BY4741 *SSA4-GFP*::*HIS3*	Thermo Fisher Scientific
Ssa4-GFP *trr1∆*	Ssa4-GFP *trr1*Δ::*URA3*	This study
Ssb1-GFP	BY4741 *SSB1-GFP*::*HIS3*	Thermo Fisher Scientific
Ssb1-GFP *trr1∆*	Ssb1-GFP *trr1∆*::*URA3*	This study
Ydj1-GFP	BY4741 *YDJ1-GFP*::*HIS3*	Thermo Fisher Scientific
Ydj1-GFP *trr1∆*	Ydj1-GFP *trr1∆*::*URA3*	This study

Plasmids		
pRS415-GPD	pRS415-*GPD*	Laboratory stock
pRS415-GPD-GFP	pRS415-*GPD-GFP*	This study
pRS415	pRS415 without promoter or terminator	Laboratory stock
pRS415-TRR1	pRS415 with *TRR1*+ 1kb up CDS + 1kb Down CDS	This study
pRS415-TRR1-2CS	pRS415 with *TRR1*+ 1kb up CDS + 1kb Down CDS with C142S and C145S substitution in *TRR1*	This study
Pho88-mCh	pRS303-*PHO88-mCherry*	(D’Urso *et al.*, 2016)
HSE-*lacZ*	p*SSA3HSE-lacZ*	Laboratory stock
FFL-GFP	pRS425-*MET25-FFL-GFP*	Laboratory stock
CPY^‡^-GFP	pRH2081(*TDH3-CPY^‡^-GFP*)	(Heck *et al.*, 2010)
tGND-GFP	pRH2476 (*TDH3-tGND-GFP*)	(Heck *et al.*, 2010)
Hsp104-mCherry	pAG415-*GPD-HSP104-mCherry*	(Malinovska *et al.*, 2012)
Ub-R-KK-YFP-Su9	YCplac33-*TPI1-Ub-R-KK-YFP-Su9*	(Yu *et al.*, 2016)
Ub-V-KK-YFP-Su9	YCplac33-*TPI1-Ub-V-KK-YFP-Su9*	(Yu *et al.*, 2016)

### Yeast culture and growth assays

Strains were cultured in standard nonselective (YPD; 1% yeast extract, 2% peptone, 2% glucose) medium or selective synthetic complete medium (SC; 2% glucose) lacking amino acids for marker selection (Sunrise Science). Strains were grown at 30°C with aeration to mid-logarithmic phase unless otherwise specified. For growth curve assays, midlogue phase cells were inoculated at a low-culture density (OD_600_ = 0.01) in 100 µl fresh medium in a 96-well sterile plate and incubated with intermittent agitation in a Synergy MX Microplate reader (Biotek Instruments) at 30°C. Heat shock experiments were performed in a shaking waterbath at 39°C in glass tubes or flasks for 20 min, unless otherwise specified. For the CHX chase experiments, CHX was added at 100 µg/ml final concentration for indicated times. To monitor Hsp42-GFP dynamics during glucose starvation, midlogue phase cells were transferred to 0.02% glucose YP medium for 90 min. Proteasome inhibition was achieved via addition of 75 µM carbobenzoxy-Leu-Leu-leucinal (MG-132, MilliporeSigma) in strains additionally carrying the *pdr5∆* deletion to allow for uptake (Collins *et al.*, 2010).

### Fluorescence microscopy

Cells were wet-mounted on glass slides and imaged immediately using an Olympus IX81-ZDC inverted microscope with a 100x objective lens using fluorescence with appropriate standard filter sets. Images were captured with a Hamamatsu ORCA camera. Quantitation was done by counting at least 100 cells and dividing the number of cells containing aggregates by the total number of cells counted. For some experiments, cells containing a single, large focus were distinguished from those containing two or more visible foci.

### HSR activation using HSE *-lacZ* reporter

Activity of Hsf1 was determined by adding 50 µl of cell suspension (cells expressing the p*SSA3HSE*-*lacZ* plasmid) to 50 µl of β-Glo reagent (Promega) in a white 96-well plate. After 30 min of incubation at 30°C, luminescence was measured in a Synergy MX Microplate reader (Abrams and Morano, 2013).

### In vivo firefly luciferase refolding assay

Refolding of the heat-labile enzyme firefly luciferase was performed as described with the following modifications (Abrams and Morano, 2013). Cells bearing the plasmid pRS425-*MET25-FFL-GFP-leu2::URA3* were grown to midlogue phase in selective medium supplemented with 1 mM methionine at 30°C. Cells were washed and subcultured in medium lacking methionine to induce expression of FFL-GFP from the *MET25* promoter for 1 h. CHX was added to cultures at 100 µg/ml final concentration to halt protein synthesis. Basal FFL activity was determined by adding 10 µl of 222 nM luciferin to 100 µl of cell culture and measuring light production using a Synergy MX Microplate reader equipped with a luminometer. Cultures were shifted to 42°C for 15 min to allow heat denaturation of the FFL enzyme, and FFL activity measured after return to 30°C growth conditions for multiple time points during the recovery period.

### Proteasomal activity

Proteasomal activity was assessed using the Proteasome 20S Activity Assay Kit (MAK172-1KT; MilliporeSigma). Experiments were performed by adding 50 µl of cell suspension to 50 µl of required reagent in a white 96-well plate. After 2 h incubation at 30°C, 20S proteasome activity was measured in a Synergy MX Microplate reader. To specifically assess 26S proteasome activity, cells harboring plasmids expressing N-rule substrates Ub-R-KK-YFP-Su9 (unstable degron) or Ub-V-KK-YFP-Su9 (stable degron) were grown at 30°C to midlogue phase and then treated with 75 μM MG-132 for 2 h. Steady state levels of both model substrates was determined in WT and *trr1∆* cells by western blotting to infer relative chaperone-independent 26S proteasomal activity.

### Western blots

Proteins were isolated using glass bead lysis as described (Abrams *et al*, 2014). Protein samples were heated (65°C, 15 min) in SDS–PAGE sample-loading buffer, loaded into 10% bis-acrylamide/SDS gels and separated by electrophoresis. Separated protein samples were transferred to a polyvinylidene difluoride (PVDF) membrane, blocked in 5% nonfat dry milk and proteins detected using monoclonal anti-GFP (Roche), monoclonal anti-PGK (Invitrogen), and monoclonal anti-Ub (EMD Millipore) antibodies, followed by detection with HRP-conjugated secondary antibody (MilliporeSigma) with enhanced chemiluminescence reagent and direct photon emission imaging. Protein bands were quantitated using Image Studio Lite (LI-COR Biosciences).

### RNA isolation and qRT-PCR

Cultures were grown in 20 ml of YPD medium at 30°C, harvested at OD_600_ 0.8, centrifuged, and the cell pellet immediately frozen on dry ice. Total RNA was isolated by the hot phenol method. For qRT-PCR assays, 1 µg of RNA was converted to cDNA using the iScript cDNA synthesis kit (Bio-Rad). Relative expression of the *SSA3* and *SSA4* genes was measured by qRT-PCR using iTaq Universal SYBR Green Supermix (Bio-Rad) and calculated using standard method (Nolan *et al.*, 2006). *TAF10* was used as the normalization control gene. All experiments were conducted with three technical and either two or three biological replicates as indicated.

### Statistical analysis

Significance was determined using GraphPad QuickCalcs Welch’s unpaired *t* test calculator (GraphPad, Dotmetrics v. 9.3.0).

## Supplementary Material



## Abbreviations used:

HSR: heat shock response
INQ: internuclear quality control compartment
IPOD: insoluble protein deposit
JUNQ: juxtanuclear quality control compartment
sHSP: small heat shock protein
UPR: unfolded protein response
UPS: ubiquitin-proteasome system.

## References

[B1] Abrams JL Morano KA (2013). Coupled assays for monitoring protein refolding in *Saccharomyces cerevisiae*. *J Vis Exp* e50432. 23892247 10.3791/50432 PMC3732071

[B2] Åkerfelt M Morimoto RI Sistonen L (2010). Heat shock factors: Integrators of cell stress, development and lifespan. *Nat Rev Mol Cell Biol* *11*, 545–555. 20628411 10.1038/nrm2938 PMC3402356

[B3] Allan KM Loberg MA Chepngeno J Hurtig JE Tripathi S Kang MG Allotey JK Widdershins AH Pilat JM Sizek HJ, *et al.* (2016). Trapping redox partnerships in oxidant-sensitive proteins with a small, thiol-reactive cross-linker. *Free Radic Biol Med* *101*, 356–366. 27816612 10.1016/j.freeradbiomed.2016.10.506 PMC5154803

[B4] Ayer A Gourlay CW Dawes IW (2014). Cellular redox homeostasis, reactive oxygen species and replicative ageing in *Saccharomyces cerevisiae*. *FEMS Yeast Res* *14*, 60–72. 24164795 10.1111/1567-1364.12114

[B5] Baker HA Bernardini JP (2021). It’s not just a phase; ubiquitination in cytosolic protein quality control. *Biochem Soc Trans* *49*, 365–377. 33634825 10.1042/BST20200694 PMC7924994

[B6] Bonner JJ Ballou C Fackenthal DL (1994). Interactions between DNA-bound trimers of the yeast heat shock factor. *Mol Cell Biol* *14*, 501–508. 8264619 10.1128/mcb.14.1.501-508.1994 PMC358400

[B7] Carter Z Creamer D Kouvidi K Grant CM (2023). Sequestrase chaperones protect against oxidative stress-induced protein aggregation and [PSI+] prion formation. *bioRxiv* 2023.10.18.562867. 10.1371/journal.pgen.1011194 PMC10931478 38422160

[B8] Chae HZ Chung SJ Rhee SG (1994). Thioredoxin-dependent peroxide reductase from yeast. *J Biol Chem* *269*, 27670–27678. 7961686

[B9] Chowdhary S Kainth AS Paracha S Gross DS Pincus D (2022). Inducible transcriptional condensates drive 3D genome reorganization in the heat shock response. *Mol Cell* *82*, 4386–4399.e7. 36327976 10.1016/j.molcel.2022.10.013 PMC9701134

[B10] Collins GA Gomez TA Deshaies RJ Tansey WP (2010). Combined chemical and genetic approach to inhibit proteolysis by the proteasome. *Yeast* *27*, 965–974. 20625982 10.1002/yea.1805 PMC3566228

[B11] Dahl JU Gray MJ Jakob U (2015). Protein quality control under oxidative stress conditions. *J Mol Biol* *427*, 1549–1563. 25698115 10.1016/j.jmb.2015.02.014 PMC4357566

[B12] D’Urso A Takahashi Y-H Xiong B Marone J Coukos R Randise-Hinchliff C Wang J-P Shilatifard A Brickner JH (2016). Set1/COMPASS and Mediator are repurposed to promote epigenetic transcriptional memory. *eLife* *5*, e16691. 27336723 10.7554/eLife.16691 PMC4951200

[B13] Escusa-Toret S Vonk WIM Frydman J (2013). Spatial sequestration of misfolded proteins by a dynamic chaperone pathway enhances cellular fitness during stress. *Nat Cell Biol* *15*, 1231–1243. 24036477 10.1038/ncb2838 PMC4121856

[B14] Fra A Yoboue ED Sitia R (2017). Cysteines as redox molecular switches and targets of disease. *Front Mol Neurosci Jun* *6*:10:167. 10.3389/fnmol.2017.00167 PMC5459893 28634440

[B15] Gietz D St Jean A Woods RA Schiestl RH (1992). Improved method for high efficiency transformation of intact yeast cells. *Nucleic Acids Res* *20*, 1425. 1561104 10.1093/nar/20.6.1425 PMC312198

[B16] Grousl T Ungelenk S Miller S Ho CT Khokhrina M Mayer MP Bukau B Mogk A (2018). A prion-like domain in Hsp42 drives chaperone facilitated aggregation of misfolded proteins. *J Cell Biol* *217*, 1269–1285. 29362223 10.1083/jcb.201708116 PMC5881502

[B17] Hahn J-S Neef DW Thiele DJ (2006). A stress regulatory network for co-ordinated activation of proteasome expression mediated by yeast heat shock transcription factor. *Mol Microbiol* *60*, 240–251. 16556235 10.1111/j.1365-2958.2006.05097.x

[B18] Hanzén S Vielfort K Yang J Roger F Andersson V Zamarbide-Forés S Andersson R Malm L Palais G Biteau B, *et al.* (2016). Lifespan control by redox-dependent recruitment of chaperones to misfolded proteins. *Cell* *166*, 140–151. 27264606 10.1016/j.cell.2016.05.006

[B19] Haslbeck M Braun N Stromer T Richter B Model N Weinkauf S Buchner J (2004). Hsp42 is the general small heat shock protein in the cytosol of *Saccharomyces cerevisiae*. *EMBO J* *23*, 638–649. 14749732 10.1038/sj.emboj.7600080 PMC1271810

[B20] Heck JW Cheung SK Hampton RY (2010). Cytoplasmic protein quality control degradation mediated by parallel actions of the E3 ubiquitin ligases Ubr1 and San1. *Proc Natl Acad Sci USA* *107*, 1106–1111. 20080635 10.1073/pnas.0910591107 PMC2824284

[B21] Herman PK (2002). Stationary phase in yeast. *Curr Opin Microbiol* *5*, 602–607. 12457705 10.1016/s1369-5274(02)00377-6

[B22] Hill SM Hanzén S Nyström T (2017). Restricted access: spatial sequestration of damaged proteins during stress and aging. *EMBO Rep* *18*, 377–391. 28193623 10.15252/embr.201643458 PMC5331209

[B23] Ho C Grousl T Shatz O Jawed A Ruger-Herreros C Semmelink M Zahn R Richter K Bukau B Mogk A (2019). Cellular sequestrases maintain basal Hsp70 capacity ensuring balanced proteostasis. *Nat Commun* *10*, 1–15. 31649258 10.1038/s41467-019-12868-1 PMC6813348

[B24] Horecka J Davis RW (2014). The 50:50 method for PCR-based seamless genome editing in yeast. *Yeast* *31*, 103–112. 24639370 10.1002/yea.2992 PMC3960506

[B25] Huh WK Falvo JV Gerke LC Carroll AS Howson RW Weissman JS O’Shea EK (2003). Global analysis of protein localization in budding yeast. *Nature* *425*, 686–691. 14562095 10.1038/nature02026

[B26] Kaganovich D Kopito R Frydman J (2008). Misfolded proteins partition between two distinct quality control compartments. *Nature* *454*, 1088–1095. 18756251 10.1038/nature07195 PMC2746971

[B27] Kaya A Koc A Lee BC Fomenko DE Rederstorff M Krol A Lescure A Gladyshev VN (2010). Compartmentalization and regulation of mitochondrial function by methionine sulfoxide reductases in yeast. *Biochemistry* *49*, 8618–8625. 20799725 10.1021/bi100908v PMC3061818

[B28] Kritsiligkou P Rand JD Weids AJ Wang X Kershaw CJ Grant CM (2018). Endoplasmic reticulum (ER) stress–induced reactive oxygen species (ROS) are detrimental for the fitness of a thioredoxin reductase mutant. *J Biol Chem* *293*, 11984–11995. 29871930 10.1074/jbc.RA118.001824 PMC6078444

[B29] Kumar A Mathew V Stirling PC (2022). Nuclear protein quality control in yeast: The latest INQuiries. *J Biol Chem* *298*, 102199. 35760103 10.1016/j.jbc.2022.102199 PMC9305344

[B30] Le Moan N Clement G Le Maout S Tacnet F Toledano MB (2006). The *Saccharomyces cerevisiae* proteome of oxidized protein thiols: contrasted functions for the thioredoxin and glutathione pathways. *J Biol Chem* *281*, 10420–10430. 16418165 10.1074/jbc.M513346200

[B31] Lee HY Cheng KY Chao JC Leu JY (2016). Differentiated cytoplasmic granule formation in quiescent and non-quiescent cells upon chronological aging. *Microb Cell* *3*, 109–119. 28357341 10.15698/mic2016.03.484 PMC5349021

[B32] Lévy E Banna NE Baïlle D Heneman-Masurel A Truchet S Rezaei H Huang ME Béringue V Martin D Vernis L (2019). Causative links between protein aggregation and oxidative stress: A review. *Int J Mol Sci* *20*. 10.3390/ijms20163896 PMC6719959 31405050

[B33] Liu IC Chiu SW Lee HY Leu JY (2012). The histone deacetylase Hos2 forms an Hsp42-dependent cytoplasmic granule in quiescent yeast cells. *Mol Biol Cell* *23*, 1231–1242. 22337769 10.1091/mbc.E11-09-0752 PMC3315813

[B34] López-Mirabal HR Winther JR (2008). Redox characteristics of the eukaryotic cytosol. *Biochim Biophys Acta - Mol Cell Res* *1783*, 629–640. 10.1016/j.bbamcr.2007.10.013 18039473

[B35] Macdiarmid CW Taggart J Kerdsomboon K Kubisiak M Panascharoen S Schelble K Eide DJ (2013). Peroxiredoxin chaperone activity is critical for protein homeostasis in zinc-deficient yeast. *J Biol Chem* *288*, 31313–31327. 24022485 10.1074/jbc.M113.512384 PMC3829442

[B36] Malinovska L Kroschwald S Munder MC Richter D Alberti S (2012). Molecular chaperones and stress-inducible protein-sorting factors coordinate the spatiotemporal distribution of protein aggregates. *Mol Biol Cell* *23*, 3041–3056. 22718905 10.1091/mbc.E12-03-0194 PMC3418301

[B37] Mathew V Tam AS Milbury KL Hofmann AK Hughes CS Morin GB Loewen CJR Stirling PC (2017). Selective aggregation of the splicing factor Hsh155 suppresses splicing upon genotoxic stress. *J Cell Biol* *216*, 4027–4040. 28978642 10.1083/jcb.201612018 PMC5716266

[B38] Miller SB Ho C Winkler J Khokhrina M Neuner A Mohamed MY Guilbride DL Richter K Lisby M Schiebel E, *et al.* (2015a). Compartment-specific aggregases direct distinct nuclear and cytoplasmic aggregate deposition. *EMBO J* *34*, 778–797. 25672362 10.15252/embj.201489524 PMC4369314

[B39] Miller SB Mogk A Bukau B (2015b). Spatially organized aggregation of misfolded proteins as cellular stress defense strategy. *J Mol Biol* *427*, 1564–1574. 25681695 10.1016/j.jmb.2015.02.006

[B40] Miyata Y Rauch JN Jinwal UK Thompson AD Srinivasan S Dickey CA Gestwicki JE (2012). Cysteine reactivity distinguishes redox sensing by the heat-inducible and constitutive forms of heat shock protein 70. *Chem Biol* *19*, 1391–1399. 23177194 10.1016/j.chembiol.2012.07.026 PMC3508472

[B41] Mogk A Bukau B (2017). Role of sHsps in organizing cytosolic protein aggregation and disaggregation. *Cell Stress Chaperones* *22*, 493–502. 28120291 10.1007/s12192-017-0762-4 PMC5465027

[B42] Mogk A Ruger-Herreros C Bukau B (2019). Cellular functions and mechanisms of action of small heat shock proteins. *Annu Rev Microbiol* *73*, 89–110. 31091419 10.1146/annurev-micro-020518-115515

[B43] Morano KA Santoro N Koch KA Thiele DJ (1999). A trans-activation domain in yeast heat shock transcription factor Is essential for cell cycle progression during stress. *Mol Cell Biol* *19*, 402–411. 9858564 10.1128/mcb.19.1.402 PMC83898

[B44] Narayanaswamy R Levy M Tsechansky M Stovall GM O’Connell JD Mirrielees J Ellington AD Marcotte EM (2009). Widespread reorganization of metabolic enzymes into reversible assemblies upon nutrient starvation. *Proc Natl Acad Sci USA* *106*, 10147–10152. 19502427 10.1073/pnas.0812771106 PMC2691686

[B45] Nolan T Hands RE Bustin SA (2006). Quantification of mRNA using real-time RT-PCR. *Nat Protoc* *1*, 1559–1582. 17406449 10.1038/nprot.2006.236

[B46] O’Connell JD Tsechansky M Royal A Boutz DR Ellington AD Marcotte EM (2014). A proteomic survey of widespread protein aggregation in yeast. *Mol Biosyst* *10*, 851–861. 24488121 10.1039/c3mb70508k PMC4142438

[B47] Peffer S Gonçalves D Morano KA (2019). Regulation of the Hsf1-dependent transcriptome via conserved bipartite contacts with Hsp70 promotes survival in yeast. *J Biol Chem* *294*, 12191–12202. 31239354 10.1074/jbc.RA119.008822 PMC6690698

[B48] Rothe S Prakash A Tyedmers J (2018). The insoluble protein deposit (IPOD) in Yeast. *Front Mol Neurosci Jul* *12*:11:237. 10.3389/fnmol.2018.00237 PMC6052365 30050408

[B49] Saarikangas J Barral Y (2015). Protein aggregates are associated with replicative aging without compromising protein quality control. *eLife* *4*, e06197. 26544680 10.7554/eLife.06197 PMC4635334

[B50] Santiago A Morano KA (2022). Oxidation of two cysteines within yeast Hsp70 impairs proteostasis while directly triggering an Hsf1-dependent cytoprotective response. *J Biol Chem* *298*, 102424. 36030825 10.1016/j.jbc.2022.102424 PMC9508553

[B51] Sathyanarayanan U Musa M Bou Dib P Raimundo N Milosevic I Krisko A (2020). ATP hydrolysis by yeast Hsp104 determines protein aggregate dissolution and size in vivo. *Nat Commun* *11*, 5226. 33067463 10.1038/s41467-020-19104-1 PMC7568574

[B52] Shrivastava A Sandhof CA Reinle K Jawed A Ruger-Herreros C Schwarz D Creamer D Nussbaum-Krammer C Mogk A Bukau B (2022). The cytoprotective sequestration activity of small heat shock proteins is evolutionarily conserved. *J Cell Biol* *221*, e202202149. 36069810 10.1083/jcb.202202149 PMC9458469

[B53] Solís EJ Pandey JP Zheng X Jin DX Gupta PB Airoldi EM Pincus D Denic V (2016). Defining the essential function of yeast Hsf1 reveals a compact transcriptional program for maintaining eukaryotic proteostasis. *Mol Cell* *63*, 60–71. 27320198 10.1016/j.molcel.2016.05.014 PMC4938784

[B54] Sontag EM Morales-Polanco F Chen J-H McDermott G Dolan PT Gestaut D Le Gros MA Larabell C Frydman J (2023). Nuclear and cytoplasmic spatial protein quality control is coordinated by nuclear-vacuolar junctions and perinuclear ESCRT. *Nat Cell Biol* *25*, 699–713. 37081164 10.1038/s41556-023-01128-6 PMC12349969

[B55] Specht S Miller SBM Mogk A Bukau B (2011). Hsp42 is required for sequestration of protein aggregates into deposition sites in *Saccharomyces cerevisiae*. *J Cell Biol* *195*, 617–629. 22065637 10.1083/jcb.201106037 PMC3257523

[B56] Spector D Labarre J Toledano MB (2001). A genetic investigation of the essential role of glutathione: mutations in the proline biosynthesis pathway are the only suppressors of glutathione auxotrophy in yeast. *J Biol Chem* *276*, 7011–7016. 11084050 10.1074/jbc.M009814200

[B57] Tkach JM Glover JR (2008). Nucleocytoplasmic trafficking of the molecular chaperone Hsp104 in unstressed and heat-shocked cells. *Traffic* *9*, 39–56. 17973656 10.1111/j.1600-0854.2007.00666.x

[B58] Trott A West JD Klaić L Westerheide SD Silverman RB Morimoto RI Morano KA (2008). Activation of heat shock and antioxidant responses by the natural product celastrol: Transcriptional signatures of a thiol-targeted molecule. *Mol Biol Cell* *19*, 1104–1112. 18199679 10.1091/mbc.E07-10-1004 PMC2262981

[B59] Trotter EW Grant CM (2003). Non-reciprocal regulation of the redox state of the glutathione-glutaredoxin and thioredoxin systems. *EMBO Rep* *4*, 184–188. 12612609 10.1038/sj.embor.embor729 PMC1315827

[B60] Trotter EW Grant CM (2005). Overlapping roles of the cytoplasmic and mitochondrial redox regulatory systems in the yeast *Saccharomyces cerevisiae*. *Eukaryot Cell* *4*, 392–400. 15701801 10.1128/EC.4.2.392-400.2005 PMC549330

[B61] Trotter EW Kao CM-F Berenfeld L Botstein D Petsko GA Gray JV (2002). Misfolded proteins are competent to mediate a subset of the responses to heat shock in *Saccharomyces cerevisiae*. *J Biol Chem* *277*, 44817–44825. 12239211 10.1074/jbc.M204686200

[B62] Tye BW Churchman LS (2021). Hsf1 activation by proteotoxic stress requires concurrent protein synthesis. *Mol Biol Cell* *32*, 1800–1806. 34191586 10.1091/mbc.E21-01-0014 PMC8684711

[B63] Verghese J Abrams J Wang Y Morano KA (2012). Biology of the heat shock response and protein chaperones: budding yeast (*Saccharomyces cerevisiae*) as a model system. *Microbiol Mol Biol Rev* *76*, 115–158. 22688810 10.1128/MMBR.05018-11 PMC3372250

[B64] Wang J Sevier CS (2016). Formation and reversibility of BiP protein cysteine oxidation facilitate cell survival during and post oxidative stress. *J Biol Chem* *291*, 7541–7557. 26865632 10.1074/jbc.M115.694810 PMC4817183

[B65] Wang Y Gibney PA West JD Morano KA (2012). The yeast Hsp70 Ssa1 is a sensor for activation of the heat shock response by thiol-reactive compounds. *Mol Biol Cell* *23*, 3290–3298. 22809627 10.1091/mbc.E12-06-0447 PMC3469052

[B66] West JD Wang Y Morano KA (2012). Small molecule activators of the heat shock response: Chemical properties, molecular targets, and therapeutic promise. *Chem Res Toxicol* *25*, 2036–2053. 22799889 10.1021/tx300264x PMC3472121

[B67] Winterbourn CC Hampton MB (2008). Thiol chemistry and specificity in redox signaling. *Free Radic Biol Med* *45*, 549–561. 18544350 10.1016/j.freeradbiomed.2008.05.004

[B68] Winzeler EA Shoemaker DD Astromoff A Liang H Anderson K Andre B Bangham R Benito R Boeke JD Bussey H, *et al.* (1999). Functional characterization of the *S. cerevisiae* genome by gene deletion and parallel analysis. *Science* *285*, 901–906. 10436161 10.1126/science.285.5429.901

[B69] Yu H Gautam AKS Wilmington SR Wylie D Martinez-Fonts K Kago G Warburton M Chavali S Inobe T Finkelstein IJ, *et al.* (2016). Conserved sequence preferences contribute to substrate recognition by the proteasome. *J Biol Chem* *291*, 14526–14539. 27226608 10.1074/jbc.M116.727578 PMC4938175

[B70] Zheng X Krakowiak J Patel N Beyzavi A Ezike J Khalil AS Pincus D (2016). Dynamic control of Hsf1 during heat shock by a chaperone switch and phosphorylation. *eLife* *5*, e18638. 27831465 10.7554/eLife.18638 PMC5127643

[B71] Saccharomyces Genome Database | SGD. Available at: www.yeastgenome.org/ (accessed 6 February 2024).

