# Supplementary material for: Cytoplasmic redox imbalance in the thioredoxin system activates Hsf1 and results in hyperaccumulation of the sequestrase Hsp42 with misfolded proteins
Source: Mol Biol Cell. 2024 Mar 5;35(4):ar53. doi: 10.1091/mbc.E23-07-0296 (PMC11064659; doi:10.1091/mbc.E23-07-0296)
Supplement: Supplementary file 1 [file mbc-35-ar53-s001.pdf]

Supplemental Materials

Molecular Biology of the Cell

Goncalves *et al*.

SUPPLEMENTAL INFORMATION FOR

Cytoplasmic redox imbalance in the thioredoxin system activates Hsf1 and results in hyperaccumulation of the sequestrase Hsp42 with misfolded proteins

GONCALVES, DUY, PEFFER AND MORANO

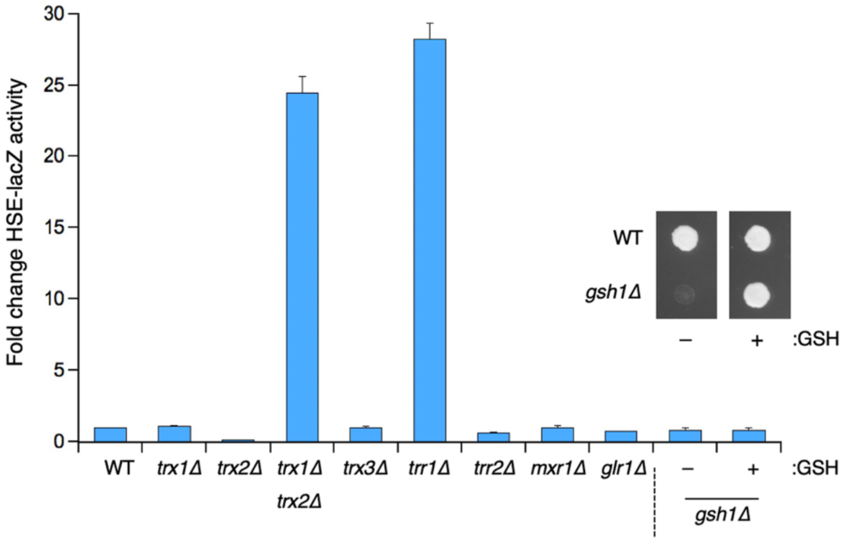

Figure S1. Chronic activation of the heat shock response occurs exclusively in cells lacking the cytosolic thioredoxin system. The indicated strains with null mutations in genes comprising different redox pathways were transformed with the p*SSA3HSE-lacZ* plasmid and basal β-galactosidase activity determined as described in Materials and Methods. Values are mean fold activation relative to wild type with error bars denoting standard deviation. To assess the HSR in *gsh1*Δ cells, reduced glutathione was added to cultures grown to mod-log phase, after which the cells were washed and diluted into glutathione-free medium and grown for an additional 6 hr prior to β-galactosidase activity determination. Inset: 3 μl aliquots of the indicated cultures were spotted onto solid medium lacking or containing 1 mM glutathione. All experiments included three biological replicates and values are the mean with error bars representing standard deviation.

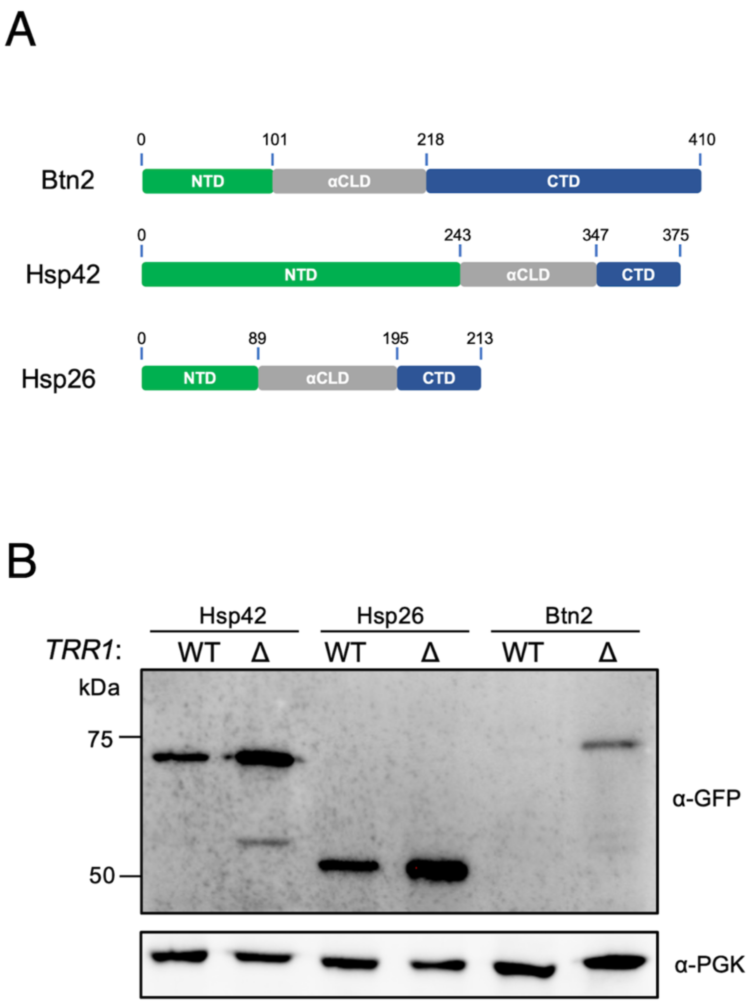

Figure S2: Expression of all three functionally related sequestrase heat shock proteins is elevated in *trr1*Δ cells. A) Domain schematics of the indicated proteins. NTD, amino terminal domain; αCLD, α-crystallin or α-crystallin-like domain; CTD, carboxyl terminal domain. B) SDS-PAGE immunoblot of protein extracts prepared from the indicated strains bearing chromosomal GFP fusions grown to mid-log phase.

A

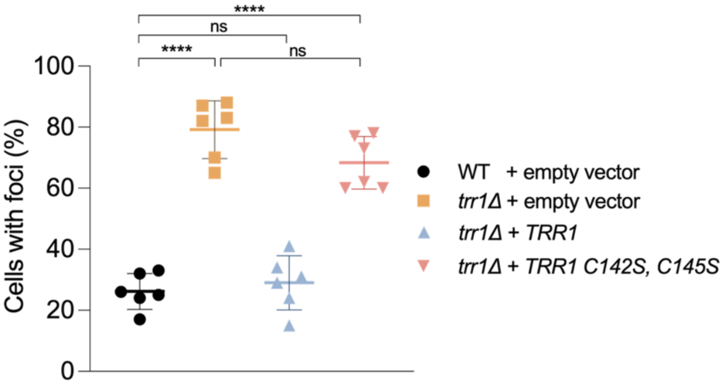

B

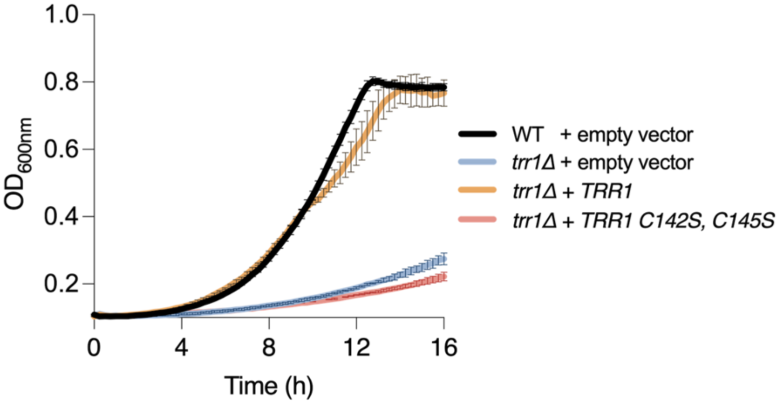

Figure S3: Thioredoxin reductase activity of Trr1 is required for normal Hsp42 spatial distribution. A) The indicated cells (WT with vector alone, or *trr1*Δ cells bearing the indicated plasmids) were grown to mid-log phase and the percentage of cells with foci was determined by counting at least 100 cells from multiple fields. B) The same strains as in (A) were inoculated at an initial OD_{600} = 0.01 in a sterile 96-well plate and grown with shaking at 30˚C for 16 h with density measurements taken every 10 min. The average of three biological replicate growth curves are shown with standard deviation. Statistical significance between the indicated strains was determined using Welch's unpaired t test (p=0.05, *; p=0.005, **; p=0.0005, ***; p=0.00005, ****)

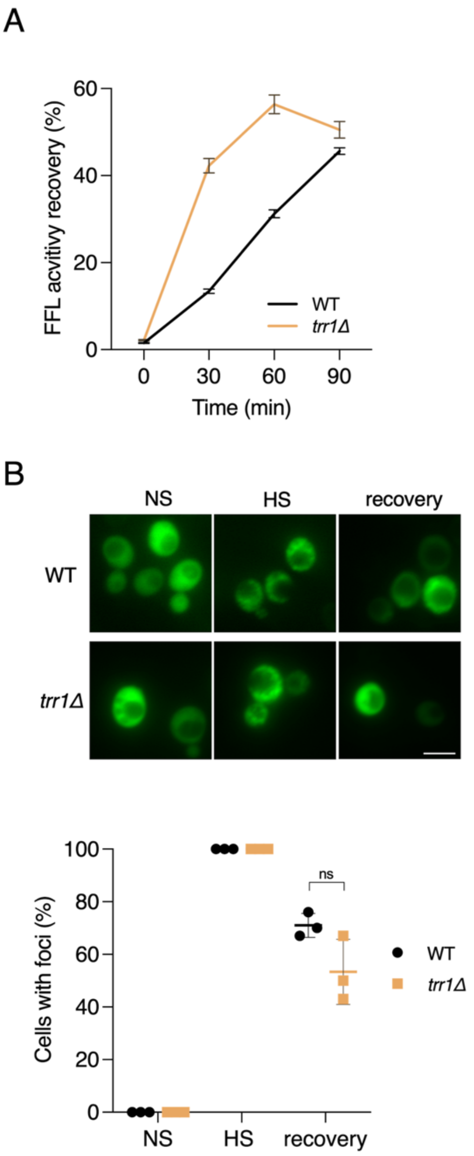

Figure S4: Folding of the model protein luciferase is not impaired in *trr1*Δ cells. A) WT or *trr1*Δ cells transformed with the FFL-GFP plasmid were grown to mid-log phase and steady state FFL activity measured as described in Materials and Methods. Cells were then heat shocked at 42˚C for 15 min to cause FFL unfolding. FFL activity in living cells was measured at the indicated time points after return to growth at 30˚C. B) Aliquots of cells from (A) were removed and imaged to identify FFL-GFP CytoQ aggregates. C) The percentage of cells in (B) with foci was determined by counting at least 100 cells from multiple fields. Scale bar = 5 μm. Statistical significance between the indicated strains was determined using Welch's unpaired t test (p=0.05, *; p=0.005, **; p=0.0005, ***; p=0.00005, ****)

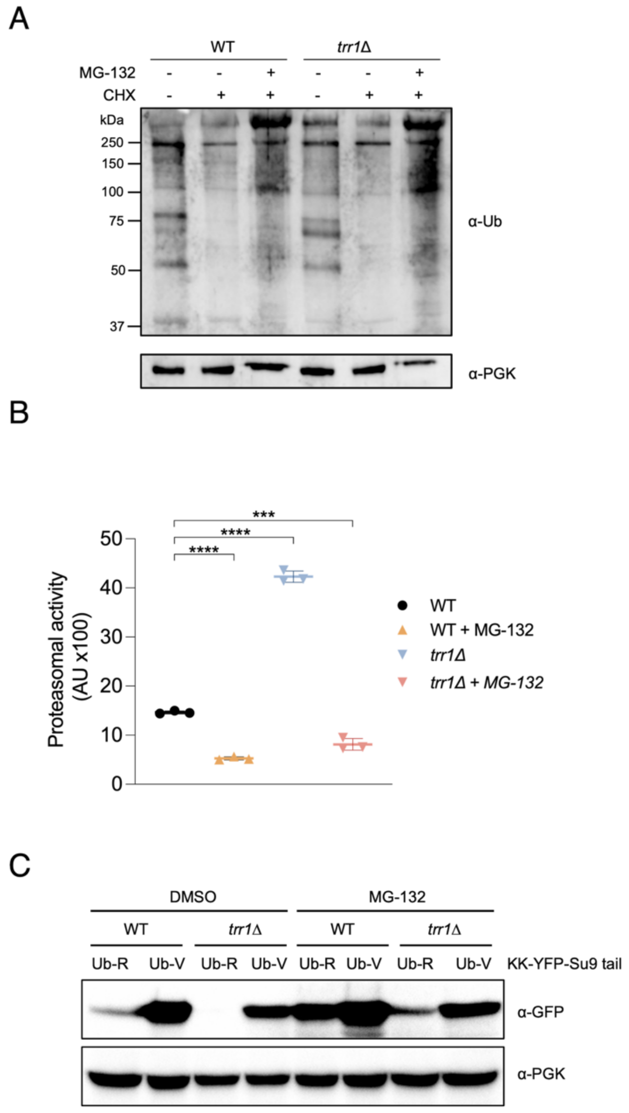

Figure S5: The ubiquitin-proteasome system is not impaired in *trr1*Δ cells. A) WT and *trr1*Δ cells (also bearing the *pdr5*Δ mutation) were grown to mid-log phase and treated or not with MG-132 at 75 μM or cycloheximide (CHX, 100 μg/ml) for 2 hr. Protein extracts were prepared, resolved by SDS-PAGE and ubiquitin profiles determined by blotting using anti-ubiquitin antibody. Levels of PGK1 were determined as a load control. B) The same strains as in (A) were grown to mid-log phase and MG-132-sensitive 20S proteasomal activity determined as described in Materials and Methods. C) WT and *trr1*Δ strains (also bearing the *pdr5*Δ mutation) harboring plasmid Ub-R-KK-YFP-Su9 or Ub-V-KK-YFP-Su9 were grown at 30°C to mid-log phase and then treated with DMSO or 75 μM MG-132 for 2 hr. Protein extracts were prepared and then subjected to western blotting. YFP fusion proteins were detected using anti-GFP antibody. Levels of PGK1 were determined as a load control. Statistical significance between the indicated strains was

determined using Welch's unpaired t test (p=0.05, *; p=0.005, **; p=0.0005, ***; p=0.00005, ****).

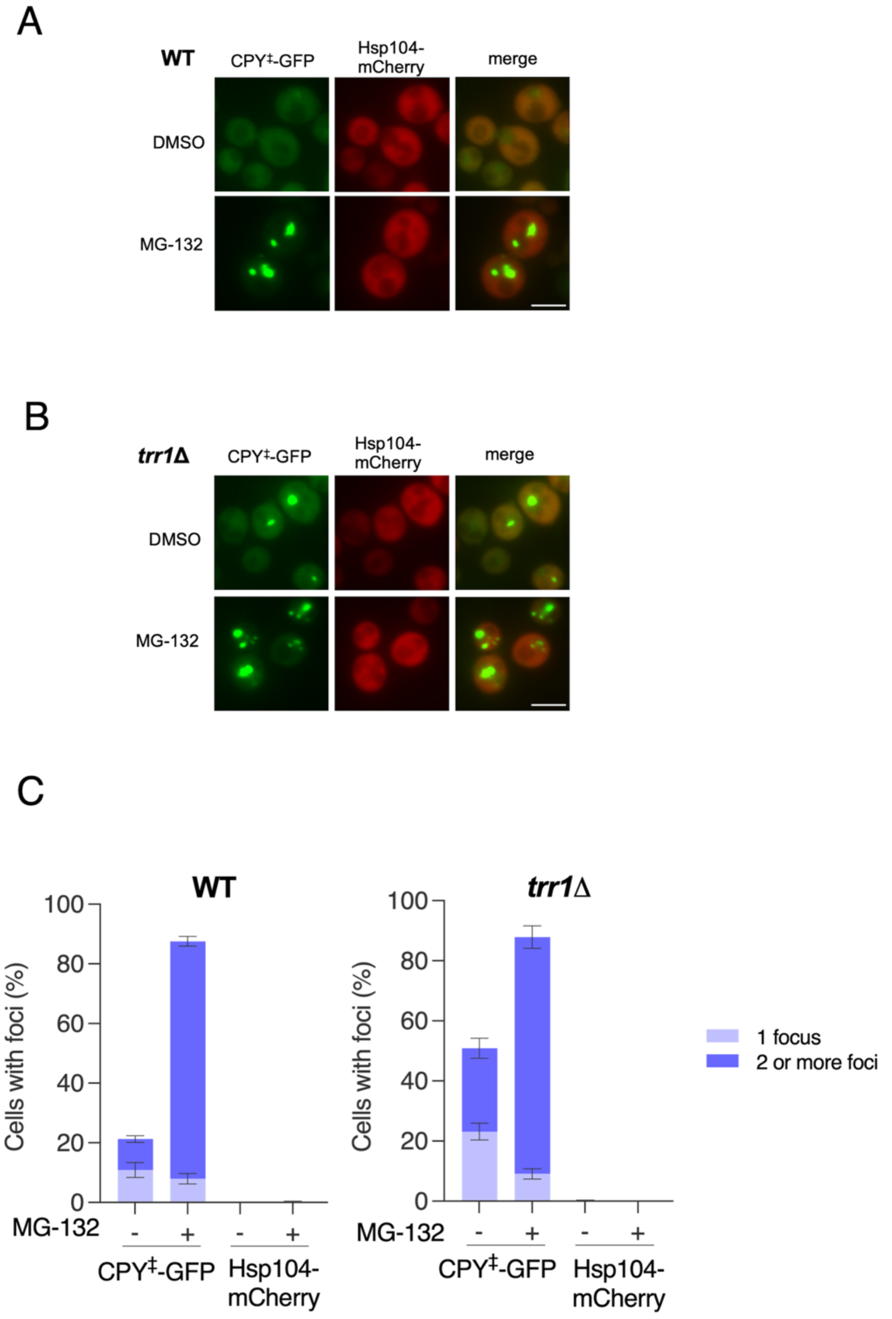

Figure S6: Hsp104 does not co-localize with CPY‡-GFP upon treatment with the proteasome inhibitor MG-132 in *trr1*Δ cells. A) *CPY‡-GFP* WT and B) *CPY‡-GFP trr1*Δ strains (additionally *pdr5*Δ) harboring the pAG415-*GPD-HSP104-mCherry* plasmid were grown at 30°C in the absence (DMSO alone) or presence of 75 μM MG-132 for 2 hr. C) The percentage of cells (mean ± SD) with either one or two or more foci was determined

by counting from three independent biological replicates. For each replicate, at least 100 cells from multiple fields were counted. Scale bar = 5 μM.
