# Supplementary material for: Cytoplasmic redox imbalance in the thioredoxin system activates Hsf1 and results in hyperaccumulation of the sequestrase Hsp42 with misfolded proteins
Source: Mol Biol Cell. 2024 Mar 5;35(4):ar53. doi: 10.1091/mbc.E23-07-0296 (PMC11064659; doi:10.1091/mbc.E23-07-0296)
Supplement: Supplementary file 2 [file reviewer_reportE23-07-0296.pdf]

Cytoplasmic redox imbalance in the thioredoxin system activates Hsf1 and results in hyperaccumulation of the sequestrase Hsp42 with misfolded proteins

Davi Gonçalves, Duong Duy, Sara Peffer, and Kevin Morano

Corresponding author(s): Kevin Morano, McGovern Medical School, UTHealth Houston

Review Timeline:

Submission Date:	2023-07-31
Editorial Decision:	2023-08-29
Revision Received:	2024-02-09
Accepted:	2024-02-10

Editor-in-Chief: Matthew Welch

Transaction Report:

(Note: With the exception of the correction of typographical or spelling errors that could be a source of ambiguity, letters and reports are not edited. The original formatting of letters and referee reports may not be reflected in this compilation.)

RE: Manuscript #E23-07-0296
TITLE: Cytoplasmic redox imbalance in the thioredoxin system activates Hsf1 and results in hyperaccumulation of the sequestrase Hsp42 with misfolded proteins

Dear Prof. Morano:

Your manuscript, titled "Cytoplasmic redox imbalance in the thioredoxin system activates Hsf1 and results in hyperaccumulation of the sequestrase Hsp42 with misfolded proteins" has been reviewed by two referees. Their verbatim comments are attached. Both referees felt that your findings, in principle, would be of interest to our MBoC readership. However, they both pointed out that the proposed mechanisms are not fully supported by experimental data. Both reviewers recommend additional experiments and changes to the manuscript that should be addressed to support and/or to tone down the authors conclusions.

Additionally, information to all relevant figure legends must be added as follow:
(1) The exact sample size (n) for each experimental group/condition. Exact numbers must be provided and not a range. I also suggest the authors to provide each data point in the form of dot plots;
(2) State if samples represent technical or biological replicates;
(3) Define error bars (s.d. or s.e.m.?).

In sum, we would be happy to consider a revised manuscript that satisfies the joint concerns of the referees. Normally, if additional experiments are needed a paper is rejected. However, these experiments can be performed relatively easily, if judged necessary to support the conclusions, and follow directly from your current results. Therefore, we look forward to receiving your revised manuscript, together with a letter indicating the changes you've made and your responses to the referees.

Sincerely,

Guillaume Thibault
Monitoring Editor
Molecular Biology of the Cell

Dear Prof. Morano,

The review of your manuscript, referenced above, is now complete. The Monitoring Editor has decided that your manuscript is not acceptable for publication at this time, but may be deemed acceptable after specific revisions are made, as described in the Monitoring Editor's decision letter above and the reviewer comments below.

A reminder: Please do not contact the Monitoring Editor directly regarding your manuscript. If you have any questions regarding the review process or the decision, please contact the MBoC Editorial Office (mboc@ascb.org).

When submitting your revision include a rebuttal letter that details, point-by-point, how the Monitoring Editor's and reviewers' comments have been addressed. (The file type for this letter must be "rebuttal letter"; do not include your response to the Monitoring Editor and reviewers in a "cover letter.") Please bear in mind that your rebuttal letter will be published with your paper if it is accepted, unless you have opted out of publishing the review history.

Authors are allowed 90 days to submit a revision. If this time period is inadequate, please contact us at mboc@ascb.org.

Revised manuscripts are assigned to the original Monitoring Editor whenever possible. However, special circumstances may preclude this. Also, revised manuscripts are often sent out for re-review, usually to the original reviewers when possible. The Monitoring Editor may solicit additional reviews if it is deemed necessary to render a completely informed decision.

In preparing your revised manuscript, please follow the instruction in the Information for Authors (www.molbiolcell.org/info-for-authors). In particular, to prepare for the possible acceptance of your revised manuscript, submit final, publication-quality figures with your revision as described.

To submit the rebuttal letter, revised manuscript, and figures, use this link: Link Not Available

MBoC offers the option to publish your work with immediate open access. Open access can increase the discoverability and usability of your research. If you would like to publish your paper with immediate open access but did not select this option during initial submission, please contact the editorial office at mbc@ascb.org.

Thank you for submitting your manuscript to MBoC. We look forward to receiving your revised paper.

Sincerely,

Eric Baker
Journal Production Manager
MBoC Editorial Office
mbc@ascb.org

Reviewer #1 (Remarks to the Author):

In the presented work the authors analyzed the connection of diverse protein quality control (PQC) components protecting cells during e.g. oxidative stress. They show that yeast cells lacking the thioredoxin reductase (Trr1) constitutively induce the Hsf1-dependent heat shock response and trigger sequestration of damaged proteins via the sHsp sequestrase Hsp42. The protein deposits forming are stable and do not attract disaggregating chaperones, thus separating them from heat-shock induced inclusions. Importantly, trr1 and hsp42 exhibit negative genetic interactions as double mutants show a strongly reduced growth rates and are more sensitive to the oxidizing compound H2O2. This indicates that Hsp42-mediated sequestration is crucial for viability of trr1 mutant cells.
The paper describes an interesting and novel link between two PQC pathways. Yet, the study also remains somewhat descriptive and premature and should be further explored as suggested below. Some drawn conclusions also do not seem to be justified. The authors are asked to address these points in a revised version of the manuscript.

Major points
Decreased activity of 26S proteasome can explain enhanced protein aggregation in trr1 cells. The authors provide evidence that protein ubiquitination and 20S activity is not restricted in trr1 cells (Fig. S5), however direct evidence for unaltered 26S activity is missing. It is suggested to monitor degradation of an N-end rule model substrate (fused to e.g. GFP) in wt and trr1 cells. This type of substrate has the advantage of not being a PQC substrate and has been frequently used in literature. This point is considered relevant as the molecular reason for enhanced sequestration of unstable CPY*-GFP in trr1 cells remains unclear.

Fig. 4B: The authors should test whether CPY*-GFP inclusions forming in yeast wt cells upon MG132 addition co-localize with Ssa1 or Hsp104. Do they show the same characteristics as inclusions forming in trr1 cells in absence of MG132?

Fig.5: Do the authors also observe synthetic growth defects upon combining trx1/2 knockouts with hsp42? It is also strongly suggested to directly test for yeast cell viabilities in the different mutant strains and under the diverse growth conditions (-/+ H2O2) by performing spot tests.

Fig. 6: The observation that Hsp42 inclusions also form during starvation or stationary phase does not necessarily support the author`s idea of a linkage between redox imbalance (in trr1 mutants) and aging/starvation. Re-addition of Glucose will cause rapid solubilization of Hsp42 foci in starved cells, but will likely not affect the stable inclusions existing in trr1 mutants. The mere occurrence of foci does not mean that they share dynamics and content. The inclusions are apparently different and respective statements should be either removed or revised.

The observation that disaggregating Ssa1 and Hsp104 do not bind and co-localize to Hsp42 inclusions in trr1 cells is surprising. Can the authors speculate why that is the case? This is an important point as heat-shock induced Hsp42 inclusions are substrates of the disaggregases.

Further criticism
Different terms have been used in the literature to describe the diverse deposition sites in yeast cells forming during stress conditions. The authors use the term JUNQ, as the inclusions forming in trr1 mutant cells are located next to the nucleus. JUNQ was re-directed by the Bukau lab to the inner nucleus (INQ) and shown to form independent of Hsp42. It might therefore be advantageous to introduce a novel term to the Hsp42-dependent inclusion in trr1 mutant cells. This is also justified as these inclusions are very stable, in contrast to JUNQ that forms only transiently upon heat shock.

Btn2 is not a sHsp and does not harbor an alpha-crystallin domain. Respective claims in the text must be corrected.

Fig. 2B: The signal intensity of e.g. Btn2-GFP does not seem to be increased in trr1 mutants, though protein levels are (Fig. S2B). Same case with Hsp26. The authors should comment on this point.

Fig. 2C: The authors should provide statistics on the juxtanuclear localization of Hsp42 inclusions. How often are they localized next to the nuclear membrane. This point is also relevant because of the diverse terminologies existing in literature (see above).

Fig. 5A: the authors should show the entire growth curves in a supplementary figure, not a cropped Y-axis

All figures: State the number of biological replicates performed in microscopy experiments. It should be at least two.

Reviewer #2 (Remarks to the Author):

In their manuscript Kevin Morano and coworkers describe the interconnection of the cytosolic thioredoxin system and the heat shock response in S. cerevisiae. The y show that in thioredoxin depleted strains the heat shock response is constitutively active and that the sequestrase Hsp42 accumulates in the juxtanuclear quality control compartment (JUNQ). Thioredoxin reductase (TRR-1) and Hsp42 depleted cells show a synthetic slow growth phenotype indicating the importance of Hsp42 under oxidative stress. Additionally, TRR-1 depleted cells show a similar distribution of Hsp42 than chronically aging and glucose-starved cells. Overall the manuscript presents a new and highly interesting but rather descriptive data set. Experiments addressing the proposed mechanisms in detail are lacking. Nevertheless, the observations are carefully interpreted and very well discussed. The data on the link between redox imbalance and chronically aging are very preliminary in the current state.

Specific comments:
- Abstract/Discussion: The abstract ends with the hypothesis on a mechanism of "long-term sequestration". Throughout the manuscript and the discussion section it gets not clear how the authors envision such a mechanism and which observations are indeed supportive and which interpretations are speculative in this context.
- The authors need to comment on the cysteine being present in Hsp42 (and on the cysteine content of Hsp26/Btn2 etc.). In the context of a mechanistically understanding of the described observations it would be highly interesting to analyze if the Hsp42-cystein is important for sequestration under oxidative stress. Is the Hsp42-cystein engaged in direct interactions with TRR-1, JUNQ , etc.? The analysis of a Hsp42-cystein-point-mutation would be highly recommended.
- Fig. 5.: The synthetic growth defect of Hsp42 and TRR-1 is very interesting. Nevertheless, controlling for Hsp26+TRR-1 synthetic growth would be appropriate to verify if it is the specific (sequestrase) function of Hsp42 under oxidative stress conditions that is impaired or if it is the promiscuous substrate-stabilizing-property of being a sHsp that is responsible for the synthetic slow growth.
- Fig. 6.: Again it remains elusive if the observations are specifically correlated to Hsp42 and if they arise from a specific function at oxidative stress or if they are indicative for a generally impaired sHsp-system. Do Hsp26 and Btn2 depleted strains still form similar JUNQ foci? It remains unclear if it is Hsp42and its specific sequestration activity at oxidative stress that is connected to the cellular energy levels, or if it might be the overall sHsp-system that is connected. Additionally, the authors need to show if the cells are still in post-diauxic phase or already completely starved in stationary phase at the described test conditions. To monitor at least one real aging parameter (CFU; mutation rate detection; apoptosis-rate) in parallel to the foci formation is necessary to strengthen the proposed redox-aging-correlation.

We were gratified to find that our study would be of interest to the MBoC readership and represents "an interesting and novel link between two PQC pathways", and that our observations were "carefully interpreted and very well discussed". Both reviewers felt the work was somewhat descriptive in nature and that we did not present mechanistic details to support our observations. While we agree that we do not yet have a full understanding of the newly discovered linkage between redox balance and misfolded protein management, we feel this study represents an important contribution that provides a foundation upon which to ask mechanistic questions going forward, as outlined in our discussion.

Below we document our responses to reviewer critiques, with our responses in blue.

Editor:

1) The exact sample size (n) for each experimental group/condition. Exact numbers must be provided and not a range.
   Defined sample sizes are now provided for each experiment in the figure legends.

2) I also suggest the authors to provide each data point in the form of dot plots;
   Due to the nature of our experiments, not all plots were appropriate to convert to dot plot format; specifically, relative fractions of cells with zero, single or multiple foci we believe are best represented as a stacked bar chart and have elected to retain that format. Figs 1A, 1B, 2A, 5A, 5B, S3A, S4B and S5B now all use the dot/line plot format with data points.

3) State if samples represent technical or biological replicates;
   All biological replicates are indicated as such in the figure legends. In the Materials and Methods, we state that each biological replicate is the average of three technical replicates where appropriate.

4) Define error bars (s.d. or s.e.m.?).
   All error bars are SD and are now defined as such in the figure legends.

Reviewer 1:

Major points

1) Decreased activity of 26S proteasome can explain enhanced protein aggregation in trr1 cells. The authors provide evidence that protein ubiquitination and 20S activity is not restricted in trr1 cells (Fig. S5), however direct evidence for unaltered 26S activity is missing. It is suggested to monitor degradation of an N-end rule model substrate (fused to e.g. GFP) in wt and trr1 cells. This type of substrate has the advantage of not being a PQC substrate and has been frequently used in literature. This point is considered relevant as the molecular reason for enhanced sequestration of unstable CPY*-GFP in trr1 cells remains unclear.
   The reviewer raises an excellent point with this observation, and in response we have done exactly as suggested, obtaining a PQC-independent N-end rule construct and a control matched construct from the Andreas Matouschek laboratory at UT Austin. These were

introduced into WT and *trr1*Δ cells and steady state levels of the respective protein fusions, in the presence or absence of the proteasome inhibitor MG-132, determined by western blot. This experiment clearly demonstrated no decrease in 26S proteasome activity and additionally suggested a modest increase, consistent with our enzymatic assay indicating enhanced 20S activity. These results are presented in newly added Fig. S5C and further cement our belief that accumulation of misfolded proteins and Hsp42 in *trr1*Δ cells is not due to lack of degradative capacity of the ubiquitin-proteasome system.

2) Fig. 4B: The authors should test whether CPY*-GFP inclusions forming in yeast wt cells upon MG132 addition co-localize with Ssa1 or Hsp104. Do they show the same characteristics as inclusions forming in trr1 cells in absence of MG132?
While we did not anticipate that Hsp104 would co-localize with CPY‡-GFP foci in *trr1*Δ cells since it did not colocalize with Hsp42-GFP foci, the reviewer raised a relevant point that prompted us to undertake an additional experiment. We obtained a functional mCherry-tagged *HSP104* allele from Simon Alberti's laboratory in Dresden and introduced it into the relevant strains and observed that as anticipated, no stable Hsp104 foci formed in WT or *trr1*Δ cells. As cited in the manuscript, the Hsp104 disaggregase is thought to transiently associate with CytoQ bodies in an ATP-dependent manner, rather than be retained as a co-chaperone in sequestrase-assembled structures. As our work focuses exclusively on chronic, steady-state scenarios as opposed to acute stress conditions, it makes sense that Hsp104 is not found to accumulate.

3) Fig.5: Do the authors also observe synthetic growth defects upon combining trx1/2 knockouts with hsp42? It is also strongly suggested to directly test for yeast cell viabilities in the different mutant strains and under the diverse growth conditions (-/+ H2O2) by performing spot tests.
We did not pursue additional genetic combinations with *HSP42* deletion after finding that the *trr1*Δ mutation was generally identical to the *trx1/2*ΔΔ knockouts for all the conditions we investigated. In addition, in our opinion the growth curves shown in Fig. 5 under normal and redox-challenged (+H_2O_2) conditions provide superior resolution relative to spot tests.

4) Fig. 6: The observation that Hsp42 inclusions also form during starvation or stationary phase does not necessarily support the author`s idea of a linkage between redox imbalance (in trr1 mutants) and aging/starvation. Re-addition of glucose will cause rapid solubilization of Hsp42 foci in starved cells, but will likely not affect the stable inclusions existing in trr1 mutants. The mere occurrence of foci does not mean that they share dynamics and content. The inclusions are apparently different and respective statements should be either removed or revised.
While we agree with the reviewer's contention that the final set of experiments presented in Fig. 6 do not conclusively link starving/aging culture with redox imbalance caused by the *trr1*Δ mutation, a great deal of literature (cited in the manuscript) has conclusively linked loss of reducing equivalents in starved or glucose-deprived cells to redox imbalance due to inability to reduce thioredoxins and downstream peroxiredoxins and cellular proteins. Consistent with Trr1 being the lynchpin between these two scenarios, our finding that glucose repletion does not restore redox balance in *trr1*Δ cells is relevant. Additionally, we tried to choose our words carefully, noting the similarities and phenotypes of the two conditions without claiming a

causal relationship yet. As we do not want to overinterpret our conclusions, we have modified the text in the results and discussion sections to soften our language and appropriately position these findings.

5) The observation that disaggregating Ssa1 and Hsp104 do not bind and co-localize to Hsp42 inclusions in trr1 cells is surprising. Can the authors speculate why that is the case? This is an important point as heat-shock induced Hsp42 inclusions are substrates of the disaggregases.
Yes, please see response to comment #2 above. Hsp104 transiently affiliates with CytoQ compartments that typically resolve over time. We do not yet understand the nature of the *trr1*Δ Hsp42 compartments, but they differ dramatically from the acute CytoQ formed in response to proteotoxic stress. In fact, one might infer that the chronic nature of the foci suggests an inability to disaggregate the components. Experiments are currently underway to gain more mechanistic insight into the nature of the *trr1*Δ-specific bodies.

6) Different terms have been used in the literature to describe the diverse deposition sites in yeast cells forming during stress conditions. The authors use the term JUNQ, as the inclusions forming in trr1 mutant cells are located next to the nucleus. JUNQ was re-directed by the Bukau lab to the inner nucleus (INQ) and shown to form independent of Hsp42. It might therefore be advantageous to introduce a novel term to the Hsp42-dependent inclusion in trr1 mutant cells. This is also justified as these inclusions are very stable, in contrast to JUNQ that forms only transiently upon heat shock.
The reviewer highlights an area of intense current interest in the field of spatial quality control. We would like to amend the observation to note that while initial work by the Frydman group identified the JUNQ compartment, Bukau's group clarified that an additional compartment they termed INQ resides on the inner face of the nuclear membrane. The field currently recognizes both compartments and in fact substrate transfer occurs between the two. Notably, nuclear-localized Btn2 appears to be crucial for INQ formation and function, and Hsp42 for the cytosolic JUNQ. We of course strongly considered naming the exaggerated compartment observed in *trr1*Δ cells, but given the number of independent names already in the literature (Hsp42 bodies, stationary phase bodies, JUNQ) and the lack of any distinguishing metabolic or component-based features, we felt it premature to do so. At the current time, we favor a more conservative approach where we can conclusively identify misfolded proteins and Hsp42 within the foci, consistent with a CytoQ or JUNQ-type structure.

7) Btn2 is not a sHsp and does not harbor an alpha-crystallin domain. Respective claims in the text must be corrected.
Work from the Bukau and Mogk groups has conclusively demonstrated that Btn2 contains an alpha-crystallin-like domain required for its misfolded protein organization activities *in vivo* and *in vitro* that was previously unrecognized due to weak sequence similarity (Nature Comms, 2019). Given that sHsps are defined mainly by the presence of such a domain, as well as the ability to nucleate assembly of misfolded proteins, the field has grown to recognize that Hsp42 and Btn2 both represent yeast sHsp homologs. However, we have amended our descriptions throughout the text to note the presence of an alpha-crystallin-like domain for accuracy.

8) Fig. 2B: The signal intensity of e.g. Btn2-GFP does not seem to be increased in trr1 mutants, though protein levels are (Fig. S2B). Same case with Hsp26. The authors should comment on this point.
Good point – we noticed this as well and while the overall levels of Btn2 remain exceedingly low even in *trr1*Δ cells as demonstrated by western blot, we were surprised not to observe a stronger signal by fluorescence microscopy. We have noted this in the text but have no good explanation for it.

9) Fig. 2C: The authors should provide statistics on the juxtanuclear localization of Hsp42 inclusions. How often are they localized next to the nuclear membrane. This point is also relevant because of the diverse terminologies existing in literature (see above).
This was a challenging experiment to do given our microscopy equipment and we did not collect quantitative data on the degree of colocalization. The image is representative of our experiments. We have softened our conclusion to reflect this qualitative result.

10) Fig. 5A: the authors should show the entire growth curves in a supplementary figure, not a cropped Y-axis.
We have redrawn the plots in Fig. 5A and 5B to reflect the full cell density ranges (note that OD represented in the microwell plate reader is not interchangeable with that measured in a standard 1 cm cuvette), as well as the full time course we measured of 36 hr.

Reviewer 2:

1) Abstract/Discussion: The abstract ends with the hypothesis on a mechanism of "long-term sequestration". Throughout the manuscript and the discussion section it gets not clear how the authors envision such a mechanism and which observations are indeed supportive and which interpretations are speculative in this context.
Our intent is to postulate that the chronic Hsp42/JUNQ structures we observe in *trr1*Δ cells are cytoprotective (based largely on the strong synthetic phenotypes shown in Fig. 5), and that they may be functionally analogous to the same structures previously observed in glucose-starved yeast cultures. Unlike the transient CytoQ bodies that form upon heat stress, or persistent JUNQ compartments that accumulate upon inhibition of the ubiquitin-proteasome system, the structures we observe persist and enlarge over time. No information is currently available to explain how this happens, we only report that it does. To ensure that we do not misrepresent the extent of our findings, we have changed the word "mechanism" to "process" in the abstract. This is more than a semantic substitution; mechanism implies that we understand how it works when we admittedly do not. But nevertheless, a process exists to manage accumulated misfolded proteins in response to redox imbalance that we intend to elucidate going forward.

2) The authors need to comment on the cysteine being present in Hsp42 (and on the cysteine content of Hsp26/Btn2 etc.). In the context of a mechanistically understanding of the described observations it would be highly interesting to analyze if the Hsp42-cystein is important for sequestration under oxidative stress. Is the Hsp42-cystein engaged in direct interactions with TRR-1, JUNQ , etc.? The analysis of a Hsp42-cystein-point-mutation would be highly recommended.

   We would very much have liked to demonstrate that the mechanism to explain altered Hsp42 localization patterns in *trr1*Δ cells is due to oxidation status of the single cysteine present in Hsp42. However, despite multiple attempts to generate a cysteine-to-alanine substitution in the chromosomal copy of *HSP42,* we have been unable to do so in the *trr1*Δ background. We believe there may be an unknown synthetic interaction complicating strain construction, and are exploring alternate ways of pursuing this obvious and important line of investigation, in addition to assessing the actual real-time redox status of the cysteine within Hsp42. We have added text to reflect the possible roles that cysteine could play in the revised Discussion.

3) Fig. 5.: The synthetic growth defect of Hsp42 and TRR-1 is very interesting. Nevertheless, controlling for Hsp26+TRR-1 synthetic growth would be appropriate to verify if it is the specific (sequestrase) function of Hsp42 under oxidative stress conditions that is impaired or if it is the promiscuous substrate-stabilizing-property of being a sHsp that is responsible for the synthetic slow growth.

   While this is an interesting postulation from the reviewer, we feel it is beyond the scope of the current investigation to comprehensively assess the contributions of the different sequestrases to growth and oxidative stress resistance of redox-imbalanced cells. A future analysis will include not just Hsp26 but Btn2 as well and will be conducted under a range of growth and environmental stress conditions.

4) Fig. 6.: Again it remains elusive if the observations are specifically correlated to Hsp42 and if they arise from a specific function at oxidative stress or if they are indicative for a generally impaired sHsp-system. Do Hsp26 and Btn2 depleted strains still form similar JUNQ foci? It remains unclear if it is Hsp42and its specific sequestration activity at oxidative stress that is connected to the cellular energy levels, or if it might be the overall sHsp-system that is connected.

   We agree with the reviewer that these are worthy questions, and they will be more fully addressed in a follow-up study.

5) Additionally, the authors need to show if the cells are still in post-diauxic phase or already completely starved in stationary phase at the described test conditions. To monitor at least one real aging parameter (CFU; mutation rate detection; apoptosis-rate) in parallel to the foci formation is necessary to strengthen the proposed redox-aging-correlation.

   At present, we are introducing the glucose depletion results as a tantalizing area for future research – we do not make claims in this manuscript distinguishing post-diauxic phase from true stationary/quiescent phase. In fact, the diauxic phase is generally considered to be the point of glucose exhaustion and switch to non-fermentative growth, which satisfies our definition of depletion of readily available reducing equivalents and matches well with

previous work from the Jakob laboratory in yeast (eLife, 2013; cited within the manuscript). We agree that additional claims regarding precise points in chronological aging would require additional data and support, but feel we are limiting our interpretations appropriately.

RE: Manuscript #E23-07-0296R
TITLE: "Cytoplasmic redox imbalance in the thioredoxin system activates Hsf1 and results in hyperaccumulation of the sequestrase Hsp42 with misfolded proteins"

Dear Prof. Morano:

I am pleased to accept your manuscript for publication in Molecular Biology of the Cell.

Thank you for revising your manuscript, entitled "Cytoplasmic redox imbalance in the thioredoxin system activates Hsf1 and results in hyperaccumulation of the sequestrase Hsp42 with misfolded proteins", in response to the reviewers' comments. I believe that the modification have improved the clarity of your manuscript. We are pleased to publish your manuscript in MBoC. Congratulations to you and your colleagues on an elegant and important study.

Sincerely,
Guillaume Thibault
Monitoring Editor
Molecular Biology of the Cell

Dear Prof. Morano:

Congratulations on the acceptance of your manuscript! Thank you for choosing to publish your work in Molecular Biology of the Cell (MBoC).

Within 10 days, an unedited PDF of your manuscript will be published on MBoC in Press, an early release version of the journal. The date your manuscript appears on this site is the official publication date.

Your copyedited and typeset manuscript will be scheduled for publication in the next available issue of MBoC. Our production team will notify you when the page proofs of your paper are ready for your review.

MBoC offers the option to publish your paper with immediate open access. Open access can increase the discoverability and usability of your research. If you would like to publish your paper with immediate open access but did not select this option during initial submission, please contact the MBoC Editorial Office (mbc@ascb.org).

We invite you to submit images related to your accepted manuscript to be considered for the journal cover.
Cover images must contain color, TIF Format
Size image at 7.4" wide x 5.85" deep
RGB and CMYK color (please submit two separate files, one in each format)
300 dpi resolution

Please also send a 1-paragraph description of the image that a general audience could understand. Include with the caption the name and institution of the individual(s) the image should be attributed to. Please provide this description in a Word file. You can send the file via email or by your favorite file transfer service to mboc@ascb.org.

Authors of Articles and Brief Communications are also encouraged to create a short video abstract to accompany their article when it is published. These video abstracts, known as Science Sketches, are up to 2 minutes long and will be published on YouTube and then embedded in the article abstract. Science Sketch Editors on the MBoC Editorial Board will guide you as you prepare your video. Information about how to prepare and submit a video abstract is available at www.molbiolcell.org/science-sketches. Please contact mboc@ascb.org if you are interested in creating a Science Sketch.

We look forward to publishing your paper in MBoC.
Sign up for MBoC's new content alerts here https://tinyurl.com/3bcsyk3e

Sincerely,

Eric Baker
Journal Production Manager
MBoC Editorial Office

mbc@ascb.org
